# Interaction of human cytomegalovirus pUL52 with major components of the viral DNA encapsidation network underlines its essential role in genome cleavage-packaging

**DOI:** 10.1128/jvi.02201-24

**Published:** 2025-03-10

**Authors:** Sarah Harmening, Boris Bogdanow, Karen Wagner, Fan Liu, Martin Messerle, Eva Maria Borst

**Affiliations:** 1 Institute of Virology, Hannover Medical School, Hannover, Germany; 2 Research group "Structural Interactomics", Leibniz Forschungsinstitut für Molekulare Pharmakologie (FMP) 28417 , Berlin, Germany; 3 Charité, Universitätsmedizin Berlin, Corporate Member of Freie Universität Berlin and Humboldt-Universität zu Berlin, Institute of Virology 522475 , Berlin, Germany; 4 Charité Universitätsmedizin Berlin 14903 , Berlin, Germany; 5 Cluster of Excellence RESIST (EXC 2155), Hannover Medical School, Hannover, Germany; 6 German Center for Infection Research (DZIF), Partner Site Hannover-Braunschweig, Hannover, Germany; University of Virginia, Charlottesville, Virginia, USA

**Keywords:** human cytomegalovirus, genome encapsidation, UL52 protein

## Abstract

Cleavage of human cytomegalovirus (HCMV) genomes and their packaging into capsids requires at least seven essential viral proteins, yet it is not completely understood how these proteins cooperate to accomplish this task. Besides the portal protein pUL104 and the terminase subunits pUL51, pUL56, and pUL89, the UL52 protein is also necessary for HCMV genome encapsidation; however, knowledge about pUL52 is scant. In the absence of pUL52, viral concatemers are not cleaved into unit-length genomes and no DNA-filled capsids are observed, yet no viral or cellular proteins interacting with pUL52 have been identified that would explain how pUL52 exerts its essential role in the HCMV infection cycle. In this study, we aimed at a comprehensive definition of pUL52-interacting proteins in infected cells. Using suitable HCMV mutants, we employed three complementary state-of-the-art proteomic approaches, namely biotin ligase-dependent proximity labeling, affinity purification, and cross-linking mass spectrometry. These experiments, combined with thorough validation by immunoblotting, pointed to several viral DNA-associated proteins and key players pivotal for genome encapsidation as interactors of pUL52. The most noticeable direct pUL52 interaction partners were the terminase subunits pUL56 and pUL89 as well as the portal protein pUL104. Hence, we suggest a model of pUL52 function in which pUL52 mediates association of HCMV genomes with the terminase subunits and the capsid portal. Taken together, our data contribute to the understanding of an essential viral process previously recognized as a prominent antiviral target. Disturbing the identified pUL52 interactions may provide a starting point to develop novel antiviral medication.

**IMPORTANCE:**

Human cytomegalovirus (HCMV) can evoke severe disease in immunocompromised patients and, moreover, is the most frequent viral cause of malformations in newborns. The virus-specific process of genome cleavage and packaging into capsids has emerged as an Achilles heel in the HCMV life cycle, which can be targeted by novel antiviral drugs, yet the mechanism of viral DNA encapsidation is only partially understood. Here, we report that the essential viral cleavage-packaging protein pUL52 interacts with several HCMV proteins known to be crucial for genome packaging, with the most prominent ones being the terminase complex and the portal protein. These data provide insight into the role of pUL52 during HCMV infection and may lay the basis for the development of additional antiviral substances tackling viral DNA packaging.

## INTRODUCTION

Human cytomegalovirus (HCMV) is the prototype member of the β-herpesviridae and possesses the largest genome of all mammalian DNA viruses, which is thought to be replicated via a rolling circle mechanism yielding branched concatemeric structures consisting of head-to-tail-linked viral genomes (1). Packaging of the 240 kbp HCMV genome into capsids shares similarities with tailed bacteriophages (2) and results in highly pressurized capsids containing densely packed nucleic acid present in a nearly crystalline state (3, 4). Genome encapsidation occurs in the cell nucleus in subnuclear structures termed replication compartments (RCs) and is driven by the terminase protein complex, a powerful molecular motor that interacts with viral DNA as well as the portal through which the genome is spooled into the preformed capsids (5). The heterotrimeric HCMV terminase is made of pUL51, pUL56, and pUL89. Following binding of the packaging signal *pac* on viral DNA, the terminase complex translocates the DNA into capsids utilizing energy originating from ATP hydrolysis (6, 7). After packaging of one genomic unit, the nuclease domain of pUL89 mediates genome cleavage (8, 9). The smallest subunit pUL51 promotes interaction of pUL56 with pUL89 and stabilizes the terminase complex (10–12).

Most likely, several copies of the terminase complex associate with the portal (13), probably mediated by the pUL56 subunit (14). The portal is built by the UL104 protein, which assembles into a dodecameric ring structure that displays substantial positional and conformational changes as genome packaging ensues (4, 15). As a result, DNA-containing C capsids are generated that eventually engage in nuclear egress governed by pUL50 and pUL53, which form the HCMV nuclear egress complex (NEC) (16, 17). Abortive DNA packaging results in empty shells called A capsids, and B capsids possibly originate from spontaneous angularization of spherical procapsids that are supposed to connect with the terminase and DNA to initiate genome encapsidation. Like A capsids, B capsids do not contain DNA, but still an inner core of scaffolding proteins, which together with the portal initiate procapsid assembly (1).

HCMV DNA cleavage-packaging has been established as an auspicious target for novel antiviral substances, as it is a virus-specific process that has no counterpart in eukaryotic cells (5, 18–20). Indeed, the approval of letermovir, a novel drug interfering with genome encapsidation (21, 22), for prophylactic treatment of patients receiving allogeneic hematopoietic stem cell transplantation to avoid HCMV reactivation has dramatically improved clinical outcome (23–25). Letermovir acts via an incompletely understood mechanism involving the HCMV terminase complex, in particular, the pUL56 subunit (26). In contrast to the previously used nucleoside analog ganciclovir, letermovir is almost without adverse side effects (24). Similarly, as observed with other anti-HCMV drugs, there is a risk of emergence of HCMV strains no longer susceptible to letermovir, especially upon inadequate administration, and *in vitro* selection experiments demonstrated that resistance maps to the three terminase subunits pUL51, pUL56, and pUL89 (27). These findings reinforce the need for additional therapeutic options to combat HCMV infection and reactivation.

Besides the terminase and the portal, at least three other viral proteins are crucial for HCMV DNA cleavage-packaging. Among those are pUL77 and pUL93, which are part of the capsid vertex-specific complex located at the portal and, with varying occupancies, at the capsid vertices (4). Moreover, pUL77 oligomers form the portal cap following genome encapsidation (4, 15, 28). In the absence of pUL77 or pUL93, HCMV genomes remain uncut and only B capsids are detected (29, 30). Likewise, an HCMV UL52 deletion mutant produces solely B capsids, and concatemeric DNA is not processed into unit-length genomes, indicating that genome packaging is not even attempted (31). pUL52 was described as a nuclear protein of approximately 75 kDa that is expressed with late kinetics (31), yet is not a structural protein incorporated into capsids or virions (32, 33). It has been found at the nuclear periphery, enclosing the RC (31), thus exhibiting a somewhat unexpected localization, as other HCMV proteins orchestrating genome encapsidation reside within the RC. The absence of pUL52 affected neither viral DNA replication nor localization of the portal protein or the terminase subunits to RC, and interaction between pUL56 and pUL89 was still observed when pUL52 was missing (31, 34). Concerning the pUL52 orthologs in α- and γ-herpesviruses, both pseudorabies virus (PRV) and herpes simplex virus type 1 (HSV-1) pUL32 were shown to be essential for viral concatemer cleavage and genome encapsidation, and HSV-1 pUL32 was proposed to modulate disulfide bond formation in several HSV-1 proteins essential for genome packaging and capsid assembly (35, 36). Kaposi's sarcoma-associated herpesvirus (KSHV) open reading frame 68 (ORF68) was shown to adopt a pentameric ring structure that binds DNA and is associated with nuclease activity (37, 38). Albeit the amino acid sequence identity between pUL52 and its orthologs is low and restricted to the C-terminal part only, all of these proteins are conserved, and it is therefore reasonable to assume a similar function for pUL52 and its HSV-1 or KSHV counterparts.

Despite its emergence as a highly suitable drug target, HCMV genome cleavage-packaging is only incompletely understood. In particular, how viral proteins governing DNA encapsidation cooperate in a concerted manner with each other remains a subject of investigation, yet knowledge of how these proteins interact and the definition of further interaction partners is still limited. This is especially true for pUL52, for which little information is available on interacting proteins, similar to HSV-1 pUL32 and KSHV ORF68. Identifying pUL52 interactors would provide a deeper insight into its crucial role in a druggable step in the HCMV life cycle, and hence facilitate the development of additional novel antivirals.

Although comprehensive studies were carried out to define herpesvirus interaction networks by applying, for instance, yeast two-hybrid experiments, assays based on fluorescence complementation, or overexpression of isolated proteins upon transient transfection, only a few viral proteins were suggested as potential pUL52 interactors (39–42), which furthermore did not emerge in all of the approaches utilized, and, importantly, interactions have not been verified in infected cells.

In this study, we therefore aimed at defining viral proteins interacting with HCMV pUL52 during infection by combining classical pull-down techniques with a proximity-dependent biotin identification (BioID) approach. To this end, HCMV mutants were generated in which the biotin ligase BioID2 is fused to pUL52. The resulting viruses were used to identify proteins getting biotinylated by or co-precipitating with pUL52 through analysis by quantitative mass spectrometry (MS), followed by their validation employing immunoblotting. The most prominent viral proteins interacting with pUL52 turned out to be the terminase subunits and the portal protein. Additionally, covalent protein-protein cross-linking indicated direct interaction between several of the identified proteins and pUL52. We therefore propose a working model in which pUL52 exerts its essential role in the HCMV infection cycle by bringing together viral DNA with the terminase complex and the portal to conduct genome cleavage-packaging.

## MATERIALS AND METHODS

### Cells and viruses

Human foreskin fibroblasts (HFFs; purchased from Merck Millipore, MA, USA) were cultivated in Dulbecco’s modified Eagle medium (PAN Biotech, Germany) supplemented with 10% fetal calf serum, 2 mM glutamine, 100 U of penicillin, and 100 µg of streptomycin sulfate per milliliter. For propagation of HFF, 20% of FibroGRO medium (Merck Millipore, #SCMF-BM) was added, while cells seeded for experiments were kept without FibroGRO. hTERT-RPE-1 cells (Clontech) were cultivated as reported elsewhere (34).

Virus mutants generated in this study are based on the HCMV laboratory strain AD169 cloned as a bacterial artificial chromosome (BAC) in *Escherichia coli* (43). Parental HCMV BAC genomes were pHD, corresponding to the original pHB5 BAC (43) but lacking ORFs UL1 to UL10 (31), the HCMV-GFP BAC derived of pHB5 and expressing the EGFP (enhanced green fluorescent protein) gene under control of the HCMV major immediate early promoter inserted into the US region (44), and the AD169-BAC pHB15, a derivative of pHB5 in which the BAC vector is flanked with *loxP* sites and ORFs US2-6 re-inserted (45).

### Plasmids

To construct pUC-BioID-Kn, the BioID2 sequence was amplified from plasmid MCS-13x Linker-BioID2-HA (Addgene, Cambridge, MA, USA; #80899) using primer pair pUC-BioID.for and pUC-BioID.rev (see Table S1 for all primer sequences). The resulting PCR product was cloned into pUC19 cut with BamHI and HindIII applying Gibson Assembly according to the manufacturer’s instructions (NEB, MA, USA), giving rise to pUC19-BioID2. To insert a kanamycin resistance marker with an I-SceI restriction site, needed for *en passant* mutagenesis, the respective sequences were amplified from pori6K-RIT (46) using primers BioID-Kn.for and BioID-Kn.rev and cloned into the PstI site of pUC19-BioID2, yielding pUC-BioID-Kn. Plasmids encoding the UL52 ORF fused to either a Strep-Flag (SF) tag or to monomeric EGFP (mGFP) are based on pOri6K-UL52-NHA-P, which provides 530 bp of sequences upstream of the UL52 ORF as promoter region (31). First, pOri6K-52P-EV was generated by QuickChange site-directed mutagenesis utilizing oligonucleotides 6K-52RFP.rev and 6K-52RFP.for (providing an EcoRV restriction site; Table S1) and pOri6K-UL52-NHA-P as template. To insert the SF tag, a PCR product was generated with primer pair UL52-SF.for and UL52-SF.rev (Table S1) and pDEST/N-SF-TAP (47) as template and cloned into the EcoRV site of pOri6K-52P-EV, resulting in pOri6K-UL52-SF. For fusion of mGFP to UL52, primers UL52-mGFP.for and UL52-mGFP.rev (Table S1) were used to amplify the mGFP ORF from pmGFP (33), and the PCR product obtained was ligated to pOri6K-52P-EV treated with EcoRV, giving rise to pOri6K-UL52-mGFP. Plasmids for transient transfections are based on pcDNA3(+). ORFs encoding UL51, UL52, or UL104 were PCR-amplified from HCMV BAC HD-UL52-SF using primer pairs UL51-HA.for and UL51-HA.rev, UL52-SF-insert.for and UL52-SF-insert.rev, and UL104.for and UL104.rev, followed by ligation to the HindIII/NotI-treated vector by Gibson Assembly. Plasmids pLA44 and pLA45 expressing UL56 or UL89 were reported earlier (11), and pmGFP-C1 was described recently (33). The integrity of all plasmids was verified by sequencing.

### BAC mutagenesis and virus reconstitution

To generate the BAC pHS-UL52-B2 in which the BioID2 biotin ligase together with a hemagglutinin (HA) epitope is fused to the N-terminus of pUL52, the corresponding sequences were amplified from pUC-BioID-Kn using primers UL52-BioID2.for and UL52-BioID2.rev (Table S1). The resulting PCR product was recombined with the HCMV-GFP BAC employing *en passant* mutagenesis in the recombination-proficient *E. coli* strain GS1783 (48, 49). Similarly, BAC pHS-B2 in which ORF UL16 is replaced by BioID2 fused to a nuclear localization signal (NLS) was constructed by *en passant* mutagenesis utilizing primer pair BioID2-NLS-Kon.for and BioID2-NLS-Kon.rev with pUC-BioID-Kn as template and HCMV-GFP as backbone. The insertion of *Cre* recombinase-encoding sequences into the AD169-BAC pHB15 (45) having the BAC vector flanked by *loxP* sites, yielding pHB15-Cre, was done analogous as reported recently for a TB40/E-derived HCMV BAC genome (46). pHB15-Cre then served as the backbone for pHBC-UL52-B2 and pHBC-B2, which express either the BioID2-UL52 fusion protein or BioID2 only and were generated in the same way as described above for pHS-UL52-B2 and pHS-B2. BACs pHD-UL52-mGFP and pHD-UL52-SF are based on pHD (having ORFs UL1-10 replaced by an Flp recognition target [FRT] site) and were generated by *Flp*-mediated recombination (50) in an identical manner as previously published UL52 mutants (31). First, the UL52 ORF was disrupted in pHD as described before (31), resulting in pHD-ΔUL52, followed by insertion of plasmid pOri6K-UL52-mGFP or pOri6K-UL52-SF into the FRT site remaining after UL1-10 deletion. An overview of HCMV mutants generated in this study is given in Table 1. Successful mutagenesis was confirmed by restriction analysis and sequencing of the relevant regions in the recombinant BACs. To reconstitute virus mutants, HFFs were transfected with the respective BACs following an established protocol (34).

**TABLE 1:** HCMV mutants generated in this study^*a*^

Name	Parental BAC	Tag/epitope/transgene	Genomic position	Application
pHS-UL52-B2	HCMV-GFP (44)	UL52 fused to HA-BioID2	Original UL52-locus	BioID assay, HA-IP
pHS-B2	HCMV-GFP (44)	NLS-BioID2	UL16 replacement	BioID assay
pHB15-Cre	AD169-BAC (45)	Cre recombinase to excise Cre and BAC vector	Adjacent to BAC vector	Backbone for pHBC mutants
pHBC-UL52-B2	pHB15-Cre	UL52 fused to HA-BioID2	Original UL52-locus	BioID assay, HA-IP
pHBC-B2	pHB15-Cre	NLS-BioID2	UL16 replacement	BioID assay
pHD-UL52-SF	pHD (31)	UL52 fused to Strep-Flag tag (47)	UL1-10 replacement	Streptactin pull-down
pHD-UL52-mGFP	pHD (31)	UL52 fused to mGFP	UL1-10 replacement	Immunofluorescence

^*a*^ HCMV BAC mutants were constructed as outlined in Materials and Methods by homologous recombination in *E. coli*, and virus was reconstituted following BAC transfection of HFF cells. HCMV-GFP, BAC-cloned AD169 genome pHB5 (43) having US2-6 replaced by the BAC vector sequences and US7-11 by EGFP under control of the HCMV MIEP; AD169-BAC, pHB5-based HCMV BAC genome having US2-6 re-inserted and the BAC vector flanked by *loxP* sites (also termed pHB15); pHB15-Cre, intron-containing *Cre* recombinase inserted directly adjacent to BAC vector; pHD, pHB5-based BAC genome with a deletion of UL1-10 ORFs.

### HA and Streptactin pull-down

For HA and Streptactin pull-down, 2.5 × 10^6^ HFFs were seeded per T75 flask. The next day (16 h prior to infection), the cell culture medium was replaced by serum-free medium. Cells were then infected at a multiplicity of infection (MOI) of 0.4 with virus mutants HS-UL52-B2 or HCMV-GFP as control (for HA pull-down), or HD-UL52-SF or the HD control virus (for Streptactin pull-down), followed by centrifugal enhancement (950 × *g* for 30 min, 20°C). Four to 6 h later, the inocula were replaced by complete medium, and cells were cultivated until day 4 postinfection (p.i.). For harvesting, cells were trypsinized and pelleted by centrifugation, and the cell pellet was washed with cold phosphate buffered saline (PBS) and resuspended in 600 µL ice-cold lysis buffer (10 mM HEPES, 150 mM NaCl, 3 mM MgCl_2_, 5 mM EDTA, 0.5% Nonidet-P40 substitute; pH 7.4, containing protease inhibitors). The cell lysates were incubated on ice for 15 min and additionally at −80°C for 1 h. To reduce the viscosity of the cell lysates, benzonase nuclease was added (125 U/mL), and samples were incubated for 30 min at 4°C on a tumbling wheel. Benzonase activity was verified by evaluating sample viscosity by pipetting and by digestion of pUC19 DNA in the presence of cell lysate (Fig. S1A). Benzonase was inactivated by the addition of EDTA (final concentration 20 mM), and the insoluble fraction was pelleted by centrifugation (30 min, 4°C, 18,500 × *g*). Five to 10% of the cell lysates was taken as input samples, mixed with 4× Roti-Load 1 solution (Carl Roth, Karlsruhe, Germany), and denatured at 99°C for 3 min. The remaining lysates were used for pull-down experiments by adding either 60 µL of equilibrated protein A-sepharose bead slurry and 2.5 µg of HA antibody (Cell Signaling Technology [CST], cat. no. 3724, clone C29F4) per sample for HA pull-down, or 60 µL of equilibrated Streptactin sepharose beads (IBA Lifesciences GmbH, Göttingen, Germany) for Streptactin pull-down. Samples were subsequently incubated at 4°C overnight with constant mixing. Beads were then washed twice with lysis buffer, once with lysis buffer lacking detergents, and once with PBS. Proteins enriched by HA pull-down were subjected to MS analysis, and samples obtained after Streptactin pull-down were examined by immunoblotting.

### BioID assay

HFFs were seeded in T75 flasks (2.5 × 10^6^ cells per flask) and infected the next day utilizing an MOI of 0.4 plus centrifugal enhancement as described above. For the BioID assay with subsequent MS analysis, the virus mutant HS-UL52-B2 was employed with HS-B2 as control virus, and for the BioID assay followed by immunoblotting,, the HCMV mutant HBC-UL52-B2 was used with HBC-B2 as control. Biotin (final concentration 50 µM) was added 24 h prior to lysis. Cells were harvested 4 days postinfection (dpi) in BioID lysis buffer (10 mM HEPES, 150 mM NaCl, 2 mM MgCl_2_, 0.1% SDS, 0.5% Nonidet-P40 substitute, 0.5% sodium deoxycholate; pH 7.4) containing protease inhibitors. Benzonase treatment was done with 125 U/mL at 4°C for 30 min with constant mixing, and insoluble material was removed by centrifuging for 30 min, at 4°C, at 18,500 × *g*. For samples to be evaluated by immunoblotting, excess biotin was removed before streptavidin pull-down by loading the lysates (diluted fivefold with BioID lysis buffer) on protein concentrator columns (Thermo Fisher Scientific; #88514; 3K MWCO), followed by centrifugation (4°C, 4,000 × *g*) until the original volume was achieved. Samples to be analyzed by mass spectrometry were deployed directly for streptavidin pull-down. To enrich biotinylated proteins, 60 µL of equilibrated streptavidin sepharose (GE HealthCare Bio-Sciences) was added to the samples, and incubation was done overnight at 4°C on a tumbling wheel. Beads were washed as described in Ortiz et al. (51) once with wash buffer WB1 (2% SDS) at room temperature (RT), once with WB2 (50 mM HEPES, 0.1% sodium deoxycholate, 1% Triton X-100, 500 mM NaCl, 1 mM EDTA, pH 7.5), once with WB3 (10 mM Tris-HCl, 250 mM LiCl, 0.5% Nonidet-P40 substitute, 0.5% sodium deoxycholate, 1 mM EDTA, pH 8.0), and twice with WB4 (50 mM Tris-HCl, 50 mM NaCl, pH 7.4). For MS analysis, beads were additionally washed three times with WB5 (50 mM ammonium bicarbonate, pH 8.0) before being further processed (see below), and samples to be assessed by immunoblotting were mixed with 1× Roti-Load 1 and boiled at 99°C for 3 min.

### Mass spectrometry analysis

For both HA-pull-down and BioID samples, experiments were designed in biological triplicate, with suitable specificity controls. The material was denatured in 8 M urea in 50 mM triethylammonium bicarbonate (TEAB) buffer, reduced for 30 min with 4 mM dithiothreitol, and alkylated with 40 mM chloroacetamide at ambient temperature for 30 min in the dark. Liquid chromatography-mass spectrometry (LC-MS) grade Lysyl Endopeptidase (Wako) was added at a concentration of 1:100 (wt/wt) and incubated for 2 h at RT. After that, the samples were diluted fourfold with TEAB, and trypsin (Promega) was added at 1:25 (wt/wt) and incubated overnight to digest proteins. Following this, the samples were desalted using stage tips purification employing C18 empore disks (Sigma).

Bottom-up proteomic samples were measured on an Orbitrap Fusion Tribrid instrument with a Thermo Scientific Dionex UltiMate 3000 system connected to a PepMap C-18 trap-column (0.075 × 50 mm, 3 mm particle size, 100 Å pore size; Thermo Fisher Scientific) and an in-house-packed C18 column (column material: Poroshell 120 EC-C18, 2.7 mm; Agilent Technologies) at 300 nL/min flow rate and 180 min gradient lengths. The MS1 scans were performed in the orbitrap using 120,000 resolution. The MS2 scans were acquired in the ion trap with standard automatic gain control as target settings, an intensity threshold of 1e4, and maximum injection time of 40 ms. A 1 s cycle time was set between master scans.

For data analysis, we used MaxQuant v.1.6.2.6 with standard settings, except that label-free quantification (LFQ) and intensity based absolute quantification (iBAQ) quantifications were enabled. Raw files were searched against a database of human amino acid sequences (UniProt, downloaded 2020), recombinant UL52 variants, as well as HCMV proteins (GenBank: EF999921.1) concatenated to their decoy versions. False discovery rate (FDR) was set at 1% at peptide spectrum match, protein, and modification site level. Data analysis was performed using Perseus software (52). First, potential contaminants, decoy hits, and proteins only identified by (modification) site were removed before log2-transforming LFQ values. Further filtering was performed when the protein was quantified less than three times in either experiment or control. Missing values were imputed on the basis of a normal distribution shrunken by a factor of 0.3 and downshifted by 1.8 standard deviations. Log2 fold-changes and *P*-values from a two-sided *t*-test were calculated based on these values. Hyperbolic curves were computed in Perseus software and served as cut-offs for the analysis.

Proteins specifically enriched with BioID-pUL52 (outside of hyperbolic curve shown in Fig. 3A) were assessed for overrepresentation of gene ontology terms using the metascape tool (53). Significance of overrepresentation is indicated by the negative decadic logarithmic p-value, with higher values indicating stronger enrichment. The corresponding numeric data are provided in Table S2.

### Cross-linking of protein complexes followed by MS analysis

HFFs were seeded in 25 15 cm dishes and cultivated until 90% confluence. Sixteen hours prior to infection, the medium was replaced by serum-free medium, and infection was done with HCMV mutant HD-UL52-SF, using volumes of virus-containing supernatant from HD-UL52-SF-infected cells (cleared of cellular debris by centrifugation [950 × *g*, 10 min, 4°C]) that ensured infection of all cells in the culture. The inoculum was replaced by complete medium 5 hours post infection (hpi), and HFFs were cultivated for another 4 days. For Streptactin pull-down, harvesting was performed as described above in a total of 12.5 mL lysis buffer. Pre-clear was performed using 200 µL of agarose beads (Thermo Fisher Scientific, cat. no. 26150) for 3 h at 4°C with constant mixing on a tumbling wheel, and pull-down was done for 3 h at 4°C by adding 200 µL of equilibrated Streptactin beads to the cleared lysate. The Streptactin matrix, including bound protein complexes, was washed as outlined above and resuspended in 500 µL cross-linking buffer (20 mM HEPES, 150 mM NaCl, pH 7.8). DSSO (disuccinimidyl sulfoxide; Thermo Fisher Scientific, cat. no. A33545) was dissolved in dimethylsulfoxide (DMSO) and added to a final concentration of 0.5 mM, and cross-linking was carried out for 20 min at RT. This was repeated once, resulting in a final DSSO concentration of 1 mM. The cross-linking reaction was quenched with Tris-HCl pH 8.0 (final concentration 20 mM) for 30 min at RT, and the supernatant was removed by centrifugation of beads at 500 × *g* for 3 min. The precipitated protein material was stored at −80°C before being prepared for cross-link proteomics sample preparation and digested as described above. The peptide mixture was desalted by using Sep-Pak C8 cartridges (Waters).

Cross-linked peptides were measured on an Orbitrap Fusion Lumos Tribrid system (Thermo Fisher Scientific) equipped with a FAIMS Pro Duo interface (Thermo Fisher Scientific) operating with Xcalibur 4.6 and Tune 4.0. For this, the sample was loaded with an online-connected Ultimate 3000 RSLC nano LC system (Thermo Fisher Scientific) onto a 50 cm analytical, in-house packed reverse-phase column (Poroshell 120 EC-C18, 2.7 µm, Agilent Technologies), and separated with a 180 min gradient going from 0.1% wt/vol formic acid (FA) in water (buffer A) to 0.1% wt/vol FA in 80% vol/vol acetonitrile (ACN) (buffer B) at a flow rate of 250 nL/min. FAIMS compensation voltages were alternated between -50,–60, and −75 V. Cross-linked peptides were detected using a stepped HCD-MS2 method, as described (54). Data analysis was performed using Scout software version (55) v.1.4.10 using default DSSO parameters with thresholds set at 1% cross-link spectrum matches-level, residue-residue-level, and protein-protein interaction-level FDR, based on target-decoy competition with reversed decoys as implemented in Scout. FDR calculations were performed separately for intra- and inter-links. For search, we used a list of protein sequences determined by bottom-up proteomics, concatenated to a list of viral protein entries of strain AD169 (5,351 entries).

### Co-immunoprecipitation (IP) of pUL56 and pUL89 from adenofected cells

To transfect hTERT-RPE-1 with BAC DNA, 1.2 × 10^6^ cells were seeded in 10 cm dishes. The next day, 3.5 µg of either pHG-ΔUL52 or pHG (31) were used for adenofection as reported elsewhere (34). Cell lysis on day 4 post-transfection and immunoprecipitation with the UL56 antibody were done as described previously (11).

### Transient transfection of HEK293T cells

The day before transfection, 3.5 × 10^6^ cells were seeded into 10 cm dishes. Fifteen micrograms of DNA in total was then employed for transfection of each dish using the jetPEI transfection reagent (Polyplus) according to the instructions of the manufacturer. Equimolar amounts of pcDNA-UL52-SF, pcDNA-UL51-HA, pcDNA-UL104, pLA44, pLA45, or pmGFP-C1 were applied in different combinations, and cells were harvested on day 2 post-transfection. Lysis was done in Streptactin lysis buffer, and further sample treatment (including benzonase digestion and Streptactin pull-down) was done as described above. Bound material was eluted by incubation at 99°C for 3 min in 1× Roti-Load 1, and samples were analyzed by immunoblotting.

### Immunofluorescence microscopy and immunoblotting

For immunofluorescence experiments, HFFs were infected at an MOI of 0.1 with pHD-UL52-mGFP or pHD as control and analyzed 4 days later as described earlier (31). Dilution of antibodies or hybridoma cell culture supernatants (see below) was as follows: anti-HA (Cell Signaling Technology, C29F4) 1:1,000, anti-pUL44 (kindly provided by Bodo Plachter, Johannes Gutenberg University Mainz, Germany) 1:100, anti-pUL56 and anti-pUL89 (10) 1:10. Secondary antibodies were Alexa Fluor 568 goat anti-mouse IgG (Invitrogen, A11031) and Alexa Fluor 568 goat anti-rabbit IgG (Invitrogen, A11036), both diluted 1:500. Confocal laser scanning microscopy was done using a Leica Inverted-3 microscope, and images were processed with the Fiji Image J software. For immunoblotting, cells were harvested 4 dpi by trypsinization and resuspended in Roti-Load 1 solution (Carl Roth, Karlsruhe, Germany) or the respective lysis buffer (see above). Antibody dilutions for immunoblotting were 1:100 for anti-pUL93 (30), anti-pUL56, and anti-pUL89, 1:200 for anti-pUL52 (10), 1:500 for anti-IE1 (Perkin Elmer, NEA-9221) and anti-IE2 (Vancouver Biotech, 12E2 mAb), 1:1,000 for anti-pUL44 and anti-BioID2 (Novus Biologicals, SS QD1), 1:2,000 for anti-glyceraldehyde 3-phosphate dehydrogenase (GAPDH; CST, 14C10), anti-pUL50, anti-pUL53 (both a kind gift of Manfred Marschall, Friedrich Alexander University Erlangen-Nürnberg, Germany), anti-pUL99 (Fitzgerald, MA, USA), anti-HA (CST C29F4), and anti-GFP (CST D5.1). Dilution for secondary antibodies anti-mouse-poly-horseradish peroxidase (HRP) and anti-rabbit-poly-HRP (Thermo Fisher Scientific) was 1:5,000, and streptavidin-HRP (Abcam) was used 1:5,000 to 1:10,000. In all experiments, the SuperSignal Western Blot Enhancer kit (Thermo Fisher Scientific, Waltham, MA) was applied according to the instructions of the manufacturer. Membranes were exposed using a ChemiDoc MP imaging system (Bio-Rad laboratories), and images were processed with Adobe Photoshop CS4 version 11.0.

## RESULTS

### HCMV mutants for biotin-based proximity labeling

**Fig 1:**
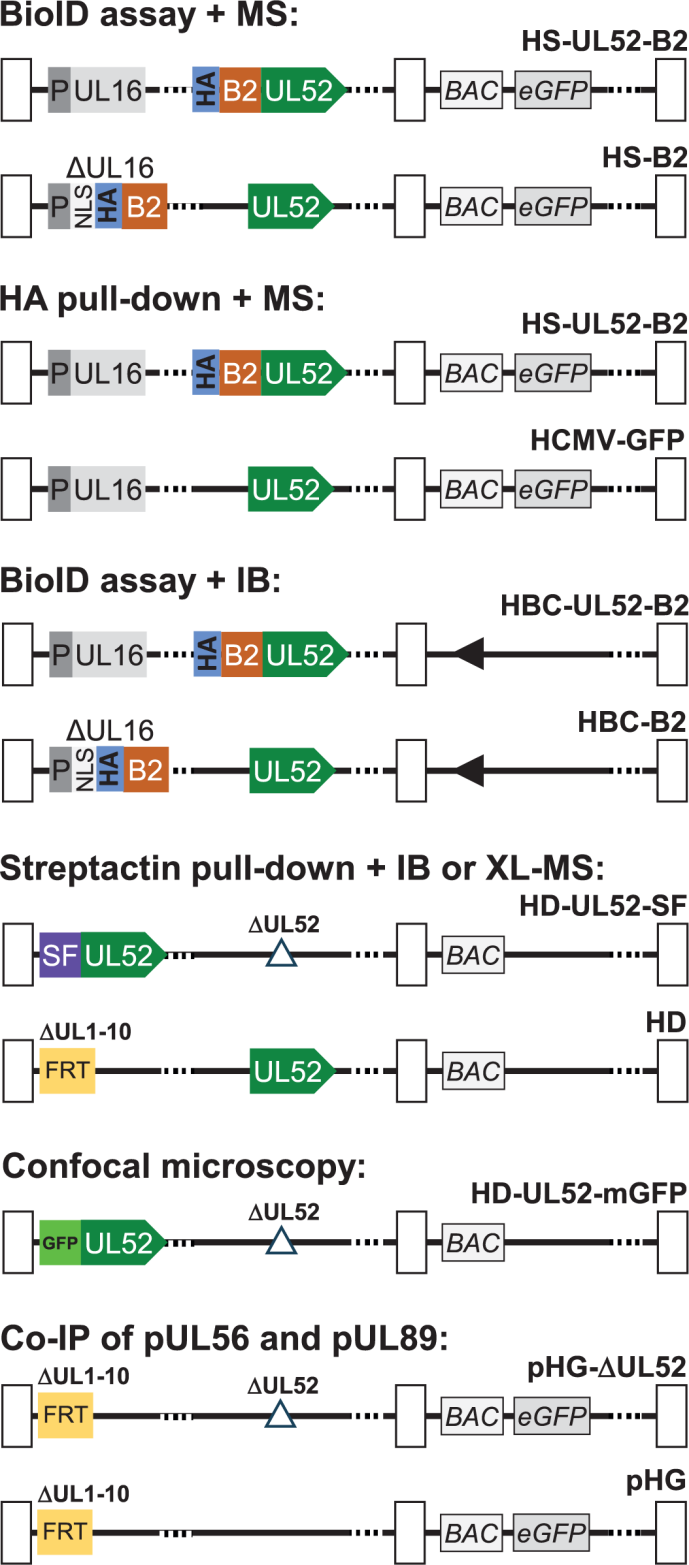
Genome structure of HCMV mutants employed in this study. For BioID proximity labeling followed by MS analysis, HS-UL52-B2 that contains UL52 fused to a BioID-HA tag was generated, and HS-B2 harboring BioID-HA with an NLS (substituting the UL16 ORF) was used as control virus. To perform HA pull-down of pUL52 with subsequent MS analysis, HS-UL52-B2 was utilized together with the published HCMV-GFP (44) as control. BioID assay evaluated by immunoblotting (IB) was done with HBC-UL52-B2 and HBC-B2 as control, which carry one *loxP* site (black rectangle) in the genome after elimination of the BAC vector. HD-UL52-SF used for Streptactin pull-down was derived from the pHD BAC in which UL1-10 is replaced by an FRT site to enable *Flp* recombinase-mediated gene insertion (31). In HD-UL52-SF, the UL52 ORF was disrupted, and UL52 fused to SF sequences was inserted via *Flp* (analogous as done for earlier HCMV UL52 mutants [31]). In the same way, HD-UL52-mGFP was constructed that expresses a fusion protein between pUL52 and monomeric EGFP. For Co-IP, the previously described BACs pHG and pHG-ΔUL52 were used (31).

**Fig 2:**
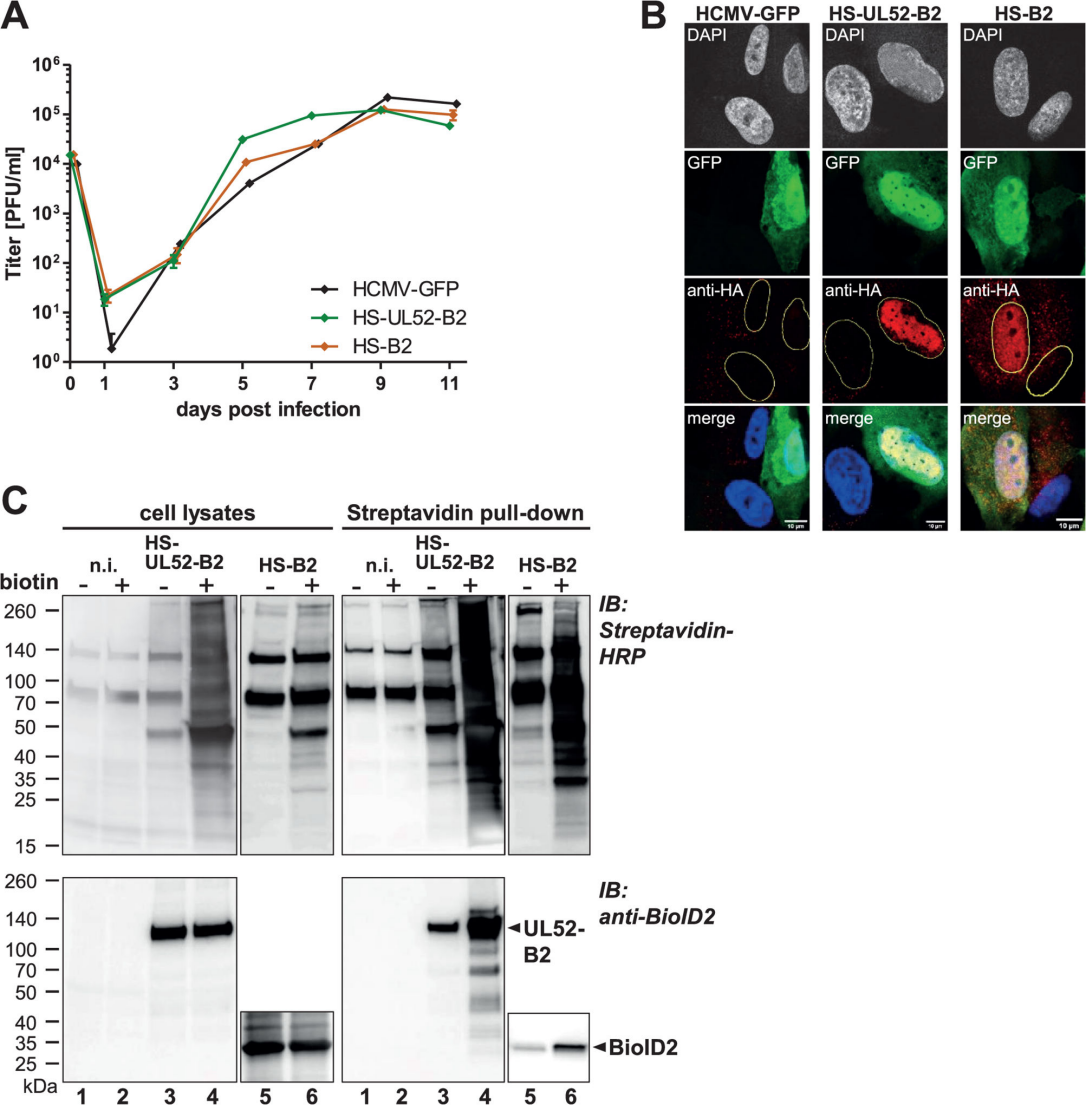
Characterization of BioID2-expressing HCMV mutants. (**A**) Growth kinetics of the HCMV BioID2 mutants and the parental virus. HFFs were infected in triplicate with an MOI of 0.1 with HS-UL52-B2 (green), HS-B2 (orange), or HCMV-GFP (black). Supernatants were collected at the indicated time points, and virus titers were determined by plaque assay. Error bars represent standard deviation. (**B**) Subcellular localization of BioID2 fusion proteins. HFFs were infected with an MOI of 0.05 with the indicated viruses, and cells were fixed 4 dpi. BioID2 fusion proteins were detected using an HA-specific antibody, and cell nuclei were visualized by DAPI (4′,6-diamidino-2-phenylindole) staining (shown in blue in the merged micrographs). Detection of EGFP served as infection control. Sample analysis was performed using confocal laser scanning microscopy. Scale bars, 10 µm. (**C**) Expression and biotinylation activity of BioID2 fusion proteins. HFFs infected with the indicated viruses were supplemented with biotin 3 dpi as indicated (+) or left untreated (−). Cells were lysed 4 dpi, and streptavidin pull-down was performed. Lysates and precipitated proteins were analyzed by immunoblotting. Biotinylated proteins were visualized with streptavidin-HRP (upper part), and BioID2 fusion proteins were detected with a BioID2-specific antibody (lower part). Expected molecular mass of UL52-B2 and BioID2 is 102 and 29 kDa, respectively.

We decided to use the proximity-dependent BioID approach to identify pUL52-interacting proteins in HCMV-infected cells. To this end, the HCMV mutant HS-UL52-B2 was generated in which the biotin ligase BioID2 (including an HA epitope) was fused to the N-terminus of pUL52 (Fig. 1; Table 1). A crucial aspect when designing proximity-labeling experiments is the specificity control, and therefore, to control for biotinylation events originating from BioID2 only, the mutant HS-B2 was constructed which has the non-essential UL16 ORF replaced by the HA-BioID2 sequences (Fig. 1; Table 1). Additionally, the BioID2 expressed by HS-B2 carries a nuclear localization signal (NLS) in order to mimic the nuclear localization of pUL52, and by using the pUL16 promoter, a similar expression kinetics as pUL52 in HCMV-infected cells was ensured (56). Both virus mutants are based on the HCMV-GFP BAC genome carrying an EGFP gene (Fig. 1) (44). As the HA-BioID2 tag added to pUL52 is relatively large in size (approximately 28 kDa), we first tested the replication properties of HS-UL52-B2 and of the control virus HS-B2 in parallel to the parental HCMV-GFP virus. The growth curve analysis showed that the HA-BioID tag did not impair growth of the mutants, and on day 11, comparable titers were reached (Fig. 2A). As expected, the UL52-B2 protein exhibited nuclear localization in HCMV-infected cells, and the same was true for the NLS-B2 fusion protein expressed by HS-B2 (Fig. 2B), demonstrating its suitability to serve as control for HS-UL52-B2. Next, we examined the recombinant viruses for expression of the BioID2-UL52 fusion protein or BioID2 alone and evaluated their biotinylation capability. HFFs were infected with the viruses HS-UL52-B2 and HS-B2 or left non-infected. Three days later, biotin was either added to the cultures or cells were left untreated for another 24 h before lysates were prepared and subjected to streptavidin pull-down to enrich for biotinylated proteins. The BioID2-UL52 fusion protein as well as BioID2 alone were readily detected in whole cell lysates as well as following pull-down using streptavidin beads (Fig. 2C, lower part, lanes 3 to 6). To assess biotinylation activity, cell lysates and pulled-down material were analyzed by immunoblotting utilizing HRP-coupled streptavidin (Fig. 2C, upper part). With non-infected HFFs, a few biotinylated proteins were observed, particularly between 70 and 140 kDa, which presumably represent cellular enzymes such as biotin-dependent carboxylases. Importantly, numerous bands that could not be resolved by SDS-PAGE were detected in lysates of HS-UL52-B2- and HS-B2-infected cells, especially after streptavidin pull-down, demonstrating strong biotinylation activity (Fig. 2C, upper right part, compare lane 3 to lane 4, and lane 5 to lane 6). These results indicate that the virus mutants HS-UL52-B2 and HS-B2 are appropriate tools to study the pUL52 interactome by BioID proximity labeling in HCMV-infected cells.

### Central factors of the viral encapsidation network are revealed by the pUL52 interactome as defined by MS

**Fig 3:**
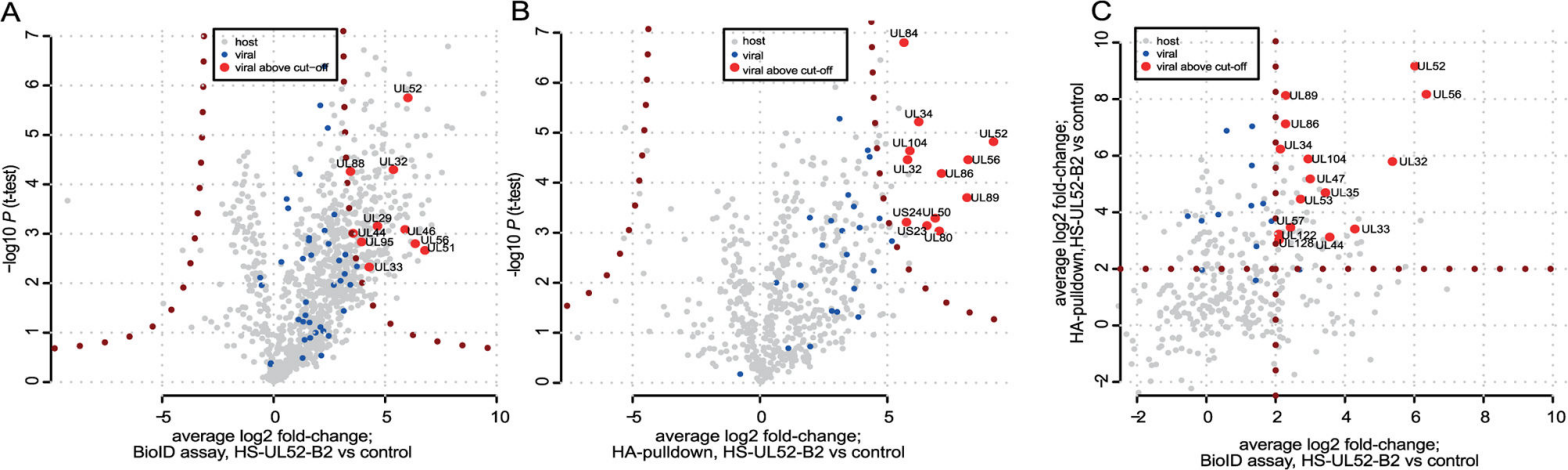
pUL52 interactome analysis by BioID assay and HA pull-down followed by MS. (**A**) HFFs were infected with the HCMV mutant HS-UL52-B2 or the control virus HS-B2. Biotin was added 3 dpi, and cells were harvested 24 h later. Biotinylated proteins were isolated using streptavidin beads, and identification and enrichment of bound proteins were carried out by MS. (**B**) HFFs infected with HS-UL52-B2 or control virus HCMV-GFP were lysed on day 4 p.i. and subjected to HA pull-down, followed by MS analysis of pUL52-interacting viral proteins. Data in (**A**) and (**B**) are depicted as volcano plot representations with average log2 fold-changes over the control and *P*-values, both on the basis of *n* = 3 biological replicates. (**C**) Comparative analysis of the MS results acquired by BioID assay and HA pull-down. *X*-axis, enrichment by BioID assay; *y*-axis, enrichment by HA pull-down. The cut-off lines indicate proteins that are greater than fourfold enriched in both assays. (**A, B, C**) Viral proteins are highlighted blue and red (when above the cut-offs).

Using the generated HCMV mutants, we performed the BioID assay followed by quantitative label-free mass spectrometry to define proteins associated with pUL52 (Fig. 3A). HFFs were infected with HS-UL52-B2 or HS-B2 as control, cell lysates were prepared on day 4 p.i. and subjected to streptavidin pull-down to enrich for biotinylated proteins. We compared protein levels in pull-down samples of HS-UL52-B2-infected fibroblasts to those of HS-B2-infected cells, the latter serving as specificity control. The enrichment ratios reflect proximity to BioID2-tagged pUL52 in intact cells during the labeling period, compared to the NLS-tagged BioID2 of the HS-B2 control virus. Overall, a range of viral and cellular proteins were enriched stronger with BioID2-pUL52, indicating that a variety of proteins was in the broader vicinity to BioID2-pUL52 during the labeling period (Fig. 3A). Among host proteins, we found many DNA-associated ones, suggesting proximity to DNA and associated protein complexes (Table S2). Gene ontology classification of these cellular proteins revealed, among others, enrichment of proteins involved in mRNA metabolism, intrinsic antiviral defense, or in the DNA damage response pathway (Fig. S1B).

As the main goal of this study was to understand the role of pUL52 in HCMV genome cleavage-packaging, we focused our analysis on viral proteins interacting with pUL52. We identified several viral candidate proteins that were reproducibly enriched (Fig. 3A, labeled in red), namely terminase constituents pUL56 and pUL51, capsid-associated proteins (triplex protein pUL46; tegument proteins pUL32, pUL88), and viral proteins involved in DNA replication and gene expression (pUL29, pUL44, pUL95) as well as pUL33 (G-protein-coupled receptor). These results indicate that during the course of the HCMV infection cycle, pUL52 comes into proximity to terminase subunits and some nucleocapsid-proximal tegument proteins as well as proteins associated with viral nucleic acids.

An advantage of the BioID2 assay is that it can reveal proteins in the broader vicinity within the intact cellular environment. This approach does, however, not indicate whether the enriched proteins are part of a stable complex. In order to comprehensively characterize the pUL52 interactome, we performed affinity purification employing the HA tag added to pUL52 in the HS-UL52-B2 mutant and the HCMV-GFP virus as specificity control. As expected for the different biochemical approaches, we observed similarities as well as differences compared to the BioID2 experiment (Fig. 3B). Again, the terminase constituent pUL56 was strongly enriched, and notably the same was true for pUL89, which is also part of the terminase complex. Likewise, tegument protein pUL32 was detected and, additionally, capsid components (pUL86: major capsid protein, and pUL80: scaffold) and the portal protein pUL104. Besides that, the NEC component pUL50, the regulatory protein pUL84, the largely uncharacterized US23 and US24 proteins, as well as pUL34 (presumably involved in capsid maturation) were observed.

Next, we directly compared the enrichment ratios of BioID and HA pull-down assays (Fig. 3C) to identify common trends between both experiments, which may be obscured by choosing strict cut-offs. This identified a range of viral proteins that are above a more loosely defined cut-off set at fourfold. In Fig. 3C, those proteins are highlighted which are enriched in each assay, i.e., which are both in vicinity to pUL52 and enriched with pUL52 following IP. We found that the terminase subunits and, to a lesser extent, the portal are both proximal and stable interactors, qualifying them as the most interesting candidates of pUL52 interaction partners (Fig. 3C). Taken together, our results suggest that pUL52 exerts its function during HCMV genome packaging through interaction with the terminase complex and likely also the portal.

### Validation of potential pUL52 interactions

We next aimed to confirm viral interactors of pUL52 identified in the MS analyses, and hence employed BioID assay and Streptactin pull-down in combination with immunoblotting using specific antibodies against the proteins of interest. For this purpose, additional HCMV mutants were generated (c.f. Fig. 1; Table 1). First, the Strep-Flag tag, successfully used already in previous studies (10–12), was fused to the N-terminus of pUL52 to enable enrichment of pUL52 and interacting proteins by Streptactin pull-down, giving rise to HCMV mutant HD-UL52-SF. Second, the genes coding for the BioID2-UL52 fusion protein as well as for BioID2 only were inserted into another AD169-derived BAC genome. Actually, during the analysis of viruses HS-UL52-B2 and HS-B2, we observed that many plaques produced by these mutants had lost the EGFP marker, whereas in the parental HCMV-GFP virus, the vast majority of plaques did express EGFP (Fig. S2A). Sequencing of the complete viral genomes revealed that in HS-UL52-B2, large regions of both the EGFP and the BAC vector sequences were deleted, and in HS-B2, the EGFP gene was lost, while other genomic regions were not affected (Fig. S2A, and data not shown). Albeit the growth kinetics of these virus mutants were similar to the parental strain (see Fig. 2A), we aimed at constructing new HCMV mutants without such issues. Murrell and colleagues have shown that HCMV BACs have a tendency to accumulate mutations and deletions in and around the prokaryotic replicon sequences, which can be obviated by making the BAC vector self-excisable (57). The additional BioID2 mutants were constructed based on the AD169-BAC/pHB15 that was derived from the original HCMV BAC pHB5 (43) by reinsertion of viral genes US2-6 and flanking of the BAC vector with *loxP* sites (45). Introduction of *Cre* recombinase sequences directly adjacent to the BAC vector was done as described recently for a TB40/E-derived BAC genome (46), followed by insertion of BioID2 analogous as described for HS-B2 and HS-UL52-B2, yielding viruses HBC-B2 and HBC-UL52-B2 (Fig. 1; Fig. S2B). PCR analyses and sequencing of the complete genomes demonstrated that in these mutants, the BAC vector and *Cre* sequences were efficiently removed via the flanking *loxP* sites, and importantly, no additional unwanted deletions were observed (Fig. S2B, and data not shown), thus indicating genomic stability of the newly generated HCMV mutants.

**Fig 4:**
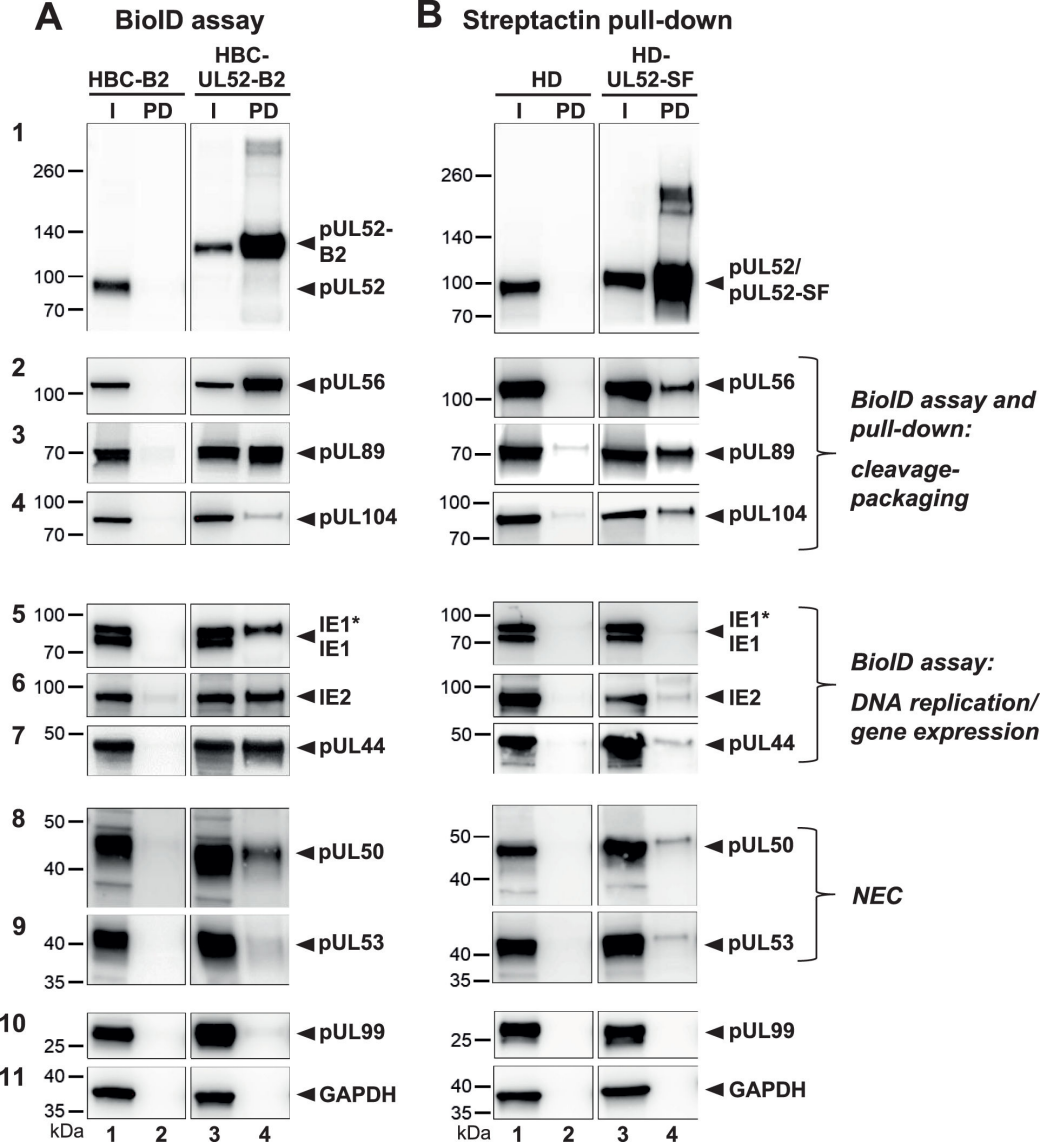
Validation of potential pUL52-interacting viral proteins. (**A**) HFFs were infected with viruses HBC-UL52-B2 or HBC-B2 (control). Biotin was added 24 h prior to cell lysis, which was done on day 4 p.i. Cell lysates (I, input) and pulled-down material (PD) were analyzed by immunoblotting with the indicated antibodies. (**B**) HFFs were infected with the viruses HD-UL52-SF or HD as control, cells were harvested 4 dpi and lysates were subjected to Streptactin pull-down. Input samples (I) and proteins bound to Streptactin beads (PD) were examined through immunoblotting. IE1*, higher molecular mass (sumoylated) IE1 variant. Faint bands detected for samples of the control viruses are considered as background signals.

The HBC-UL52-B2 and HBC-B2 mutants were then used to evaluate pUL52-interacting viral proteins by BioID assay in infected fibroblasts, followed by immunoblotting with antibodies against the putative interactors identified by MS. HFFs were infected with the respective HCMV mutants, biotin was added on day 3 p.i., and harvesting was done 24 h later. Figure 4A shows that upon infection with HBC-UL52-B2 and its control virus HBC-B2, comparable levels of the proteins of interest were present in the whole cell lysates (lanes 1 and 3). Streptavidin pull-down utilizing cells infected with virus mutant HBC-UL52-B2 demonstrated that the biotinylated UL52-B2 fusion protein was efficiently enriched (first panel, lane 4), whereas, as expected, this was not the case for pUL52 after identical treatment of lysates from HFFs infected with the control virus HBC-B2 (first panel, lane 2). The terminase subunits pUL56 and pUL89 were distinctly detected with HBC-UL52-B2 (Fig. 4A, panels 2 and 3, lane 4), while a weaker signal was obtained for the portal protein pUL104 (panel 4, lane 4). Furthermore, immediate early proteins IE1 and IE2 as well as the DNA polymerase accessory factor pUL44 were strongly biotinylated by pUL52-B2 (Fig. 4A, panels 5–7, lane 4), while concerning the NEC, one component, pUL50, but not the other one (pUL53), was moderately enriched (panel 8 and 9, lane 4). With respect to the negative controls, pUL99 (tegument protein) and the cellular GAPDH were not biotinylated in HBC-B2-infected cells (panels 10 and 11, lane 4), and none of the putative pUL52 interaction partners was identified following streptavidin pull-down from HBC-B2-infected fibroblasts (Fig. 4A, panels 2–9, lane 2). In sum, these data provide evidence that during the HCMV infection cycle, pUL52 is in close proximity to components of the genome cleavage-packaging network as well as to some DNA-associated viral proteins.

To examine whether the presumed interactors also form stable complexes with pUL52, we performed Streptactin pull-down experiments with lysates of HFFs infected with HD-UL52-SF or the parental HD virus (c.f. Fig. 1). Again, there were no major differences in expression of the proteins of interest following infection with HD-UL52-SF or the control virus HD (Fig. 4B, lanes 1 and 3). The pUL52-SF fusion protein became highly enriched by the Streptactin matrix (Fig. 4B, first panel, lane 4), whereas no signal corresponding to pUL52 was found in HD-infected HFF (Fig. 4B, first panel, lane 2). Of note, pUL56 and pUL89 as well as pUL104 did clearly co-purify with pUL52-SF (panels 2–4, lane 4), whereas the signals for the NEC components pUL50 and pUL53 (both nuclear proteins) were rather weak (panels 8 and 9, lane 4), and interaction with IE1, IE2, and pUL44, all three present in high abundance in nuclear replication compartments, was hardly detectable (Fig. 4B, panels 5–7, lane 4). Again, no or only faint background signals were observed after Streptactin pull-down from lysates of HFFs infected with the HD control virus (Fig. 4B, panels 2–9, lane 2), and neither pUL99 nor GAPDH were observed with HD- or HD-UL52-SF-infected cells (Fig. 4B, last two panels, lanes 2 and 4). As to the innermost tegument protein pUL32, which was suggested as a candidate pUL52 interactor by MS (c.f., Fig. 3C), the BioID assay may indicate proximity to pUL52, while the Streptactin pull-down argued against stable or strong interaction (Fig. S3A). In conclusion, our results indicate that pUL52 is associated with subunits of the terminase complex and probably also with the portal vertex, while biotinylation of IE1, IE2, and pUL44 rather points to spatial vicinity only.

### Verification of pUL52 interaction with the terminase and the portal protein upon transient expression

**Fig 5:**
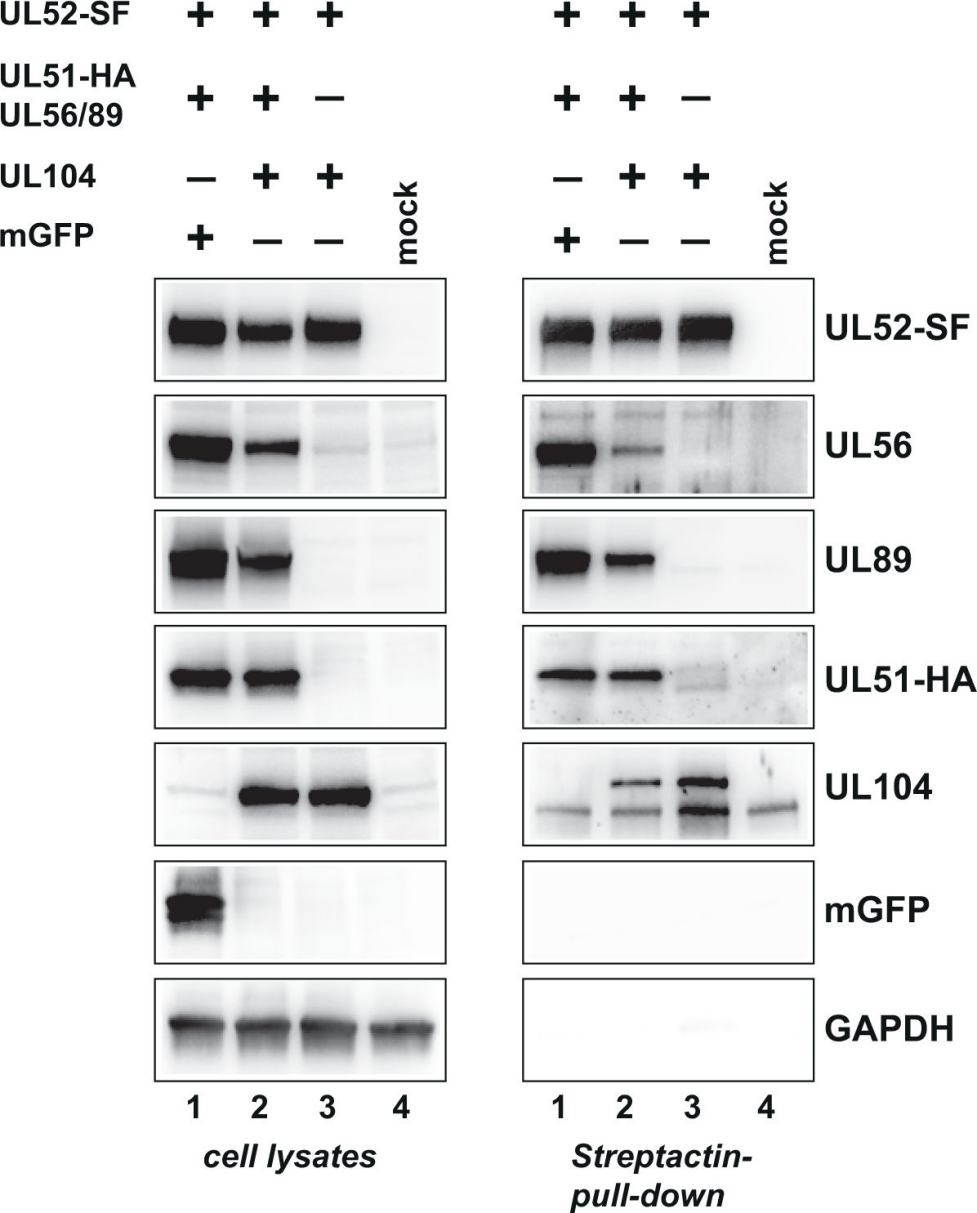
Interaction of pUL52 with the terminase subunits and the portal protein following transient expression. HEK293T cells were transfected with plasmids coding for UL52-SF, UL51-HA, UL56, UL89, UL104, or mGFP in the depicted combinations. Two days later, cell lysates were prepared and checked for the presence of the proteins of interest by immunoblotting (left). Lysates were then subjected to Streptactin pull-down, and pUL52-SF-associated proteins were analyzed by immunoblotting (right). Proteins were detected with the indicated antibodies (anti-UL52 for pUL52-SF and anti-HA for pUL51), GAPDH served as loading control.

We next asked whether the main pUL52 interactions, i.e., those with the terminase and the portal, do also occur when these proteins were expressed independent of viral infection. To this end, HEK293T cells were transfected with plasmids encoding UL52-SF, UL104, or the terminase subunits pUL56, pUL89, and pUL51-HA in different combinations. As control, a plasmid expressing monomeric EGFP was included. Following transfection, pUL52-SF was enriched by Streptactin pull-down and associated proteins were analyzed by immunoblotting. Figure 5 displays that pUL52 did interact with the terminase subunits under these conditions (right part, lane 1) and also the portal protein pUL104 (right part, lane 3), and the same results were observed when all components were expressed together (right part, lane 2). The negative control proteins (mGFP and GAPDH) were not associated with pUL52. These results show that the interactions of pUL52 with the terminase constituents and the portal protein also occur in the absence of other viral proteins and viral DNA.

### Cross-linking mass spectrometry (XL-MS) discloses direct interaction partners of pUL52

**Fig 6:**
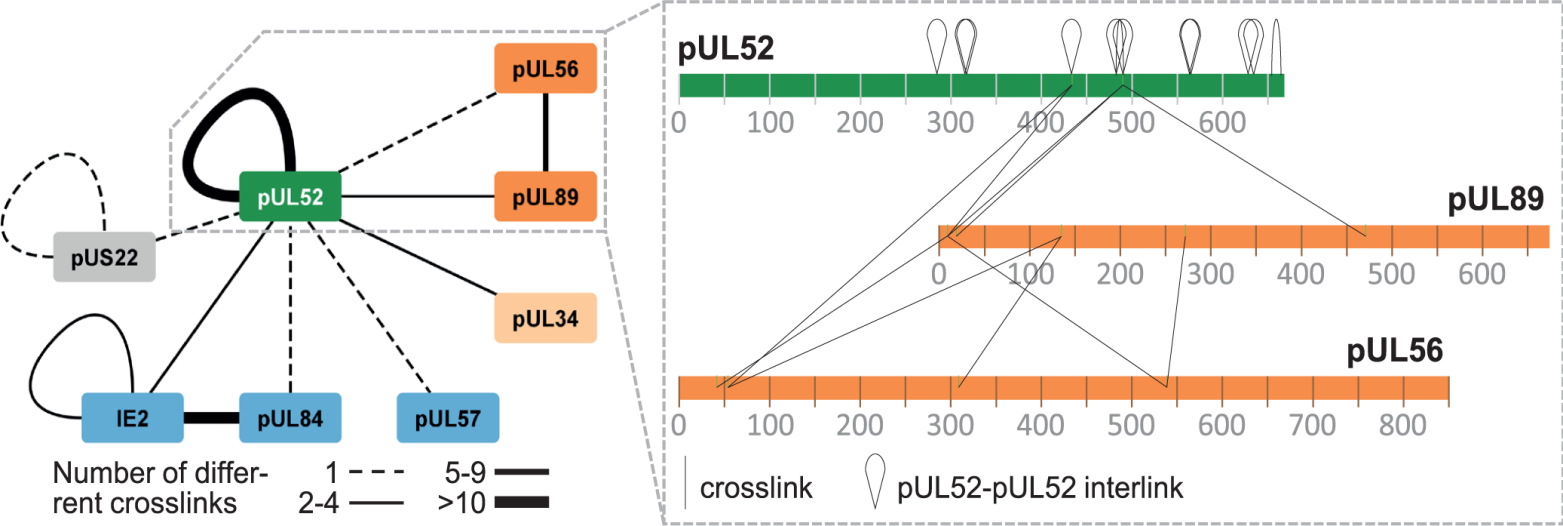
Examination of pUL52 interactions by XL-MS. HFFs infected with HD-UL52-SF were harvested 4 dpi, and lysates were subjected to Streptactin pull-down before chemical cross-linking. Nodes represent viral proteins identified by cross-links in these samples: pUL52 bait, green; terminase subunits, orange; viral DNA-binding proteins, blue; pUL34, beige, and tegument protein pUS22, gray. Solid and dashed lines correspond to the number of identified cross-links as indicated. A more detailed view shown to the right depicts the cross-links identified between pUL52 and the terminase subunits pUL56 and pUL89 as well as between the two terminase constituents and pUL52-pUL52 inter-links. Note that only cross-links between viral proteins are depicted.

Our experiments so far discovered several viral pUL52 interactors; however, neither the BioID2 assays nor the pull-down studies allowed us to conclude which of the identified proteins are direct interaction partners of pUL52. Besides that, BioID labels proteins in a vicinity of 10 nm, which may also enclose more indirectly associating proteins. In order to find out which proteins are directly binding to pUL52, we performed an XL-MS experiment using the cross-linker DSSO, which has a labeling radius of 4 nm. For this purpose, HFFs were infected with HD-UL52-SF; 4 days later, cell lysates were prepared, and pUL52-SF, including associated proteins, was enriched by Streptactin pull-down. Samples were treated with DSSO to introduce covalent cross-links into the protein complexes, followed by proteolytic digestion and analysis of the resulting peptides by MS. As summarized in Fig. 6, we observed several viral interaction partners of pUL52, with varying coverage by cross-links. Several cross-links were observed between pUL52 and pUL89 as well as between pUL52 and pUL34 or IE2. Fewer cross-links were detected between pUL52 and pUL56 as well as pUL57, pUL84, or pUS22. The lack of cross-links between pUL52 and pUL51 (third terminase subunit) can be explained by, firstly, the small size of pUL51 (157 aa), which will result in only a few peptides detectable by MS, which probably fell below the threshold. Secondly, pUL51 contains only seven lysine residues in total. Those are required to mediate cross-linking to DSSO, yet six of them are located in the C-terminus that, according to structure prediction (13), is buried within the terminase complex and is hence unlikely to be accessible to the cross-linker. Importantly, the known interactions among IE2 and pUL84 (58–60) or pUL56 and pUL89 (61–63) were identified with high confidence (≥5–10 different residue-residue connections, Fig. 6), indicating that our experimental design convincingly recapitulated previously defined interactions. Likewise, more than 10 inter-links were found with pUL52 itself (c.f., Fig. 6), indicating self-interaction or oligomerization, as was recently described for the pUL52 orthologs in α- and γ-herpesviruses (37). Taken together, these data further substantiate our findings that pUL52 interacts with components of the terminase complex.

### Subcellular localization of pUL52

**Fig 7:**
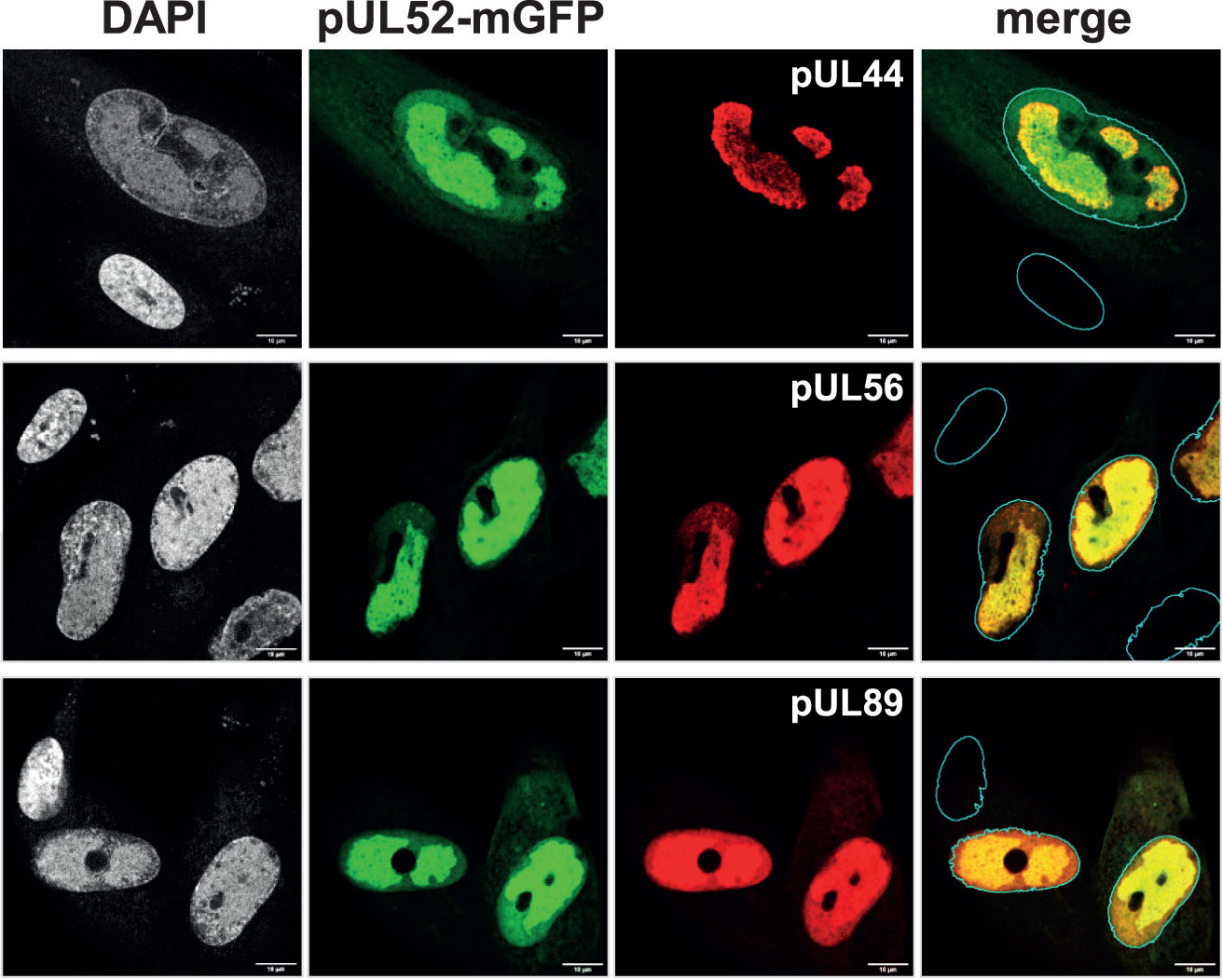
Subcellular localization of pUL52. HFFs were infected with viral mutant HD-UL52-mGFP and fixed 4 dpi. Immunolabeling and confocal laser scanning microscopy were carried out with antibodies against pUL44, pUL56, or pUL89, and nuclei were visualized via DAPI (4′,6-diamidino-2-phenylindole) staining. Scale bars, 10 µm.

Cleavage of HCMV DNA and its packaging into capsids occurs in the cell nucleus, and in line with this, the terminase and the portal accumulate in nuclear RCs of infected cells (31). In this respect, our findings on the interaction of these proteins with pUL52 were surprising, as we had previously reported that pUL52 is nuclear but located outside the RC (31). However, as depicted in Fig. 2B, staining with a different HA antibody than employed before now suggested that pUL52 is rather enriched within the viral RC. In order to gain further insight into pUL52 localization, we generated the HCMV mutant HD-UL52-mGFP in which the monomeric EGFP is fused to the N-terminus of pUL52 (Fig. 1; Table 1). This virus allows the direct detection of pUL52-mGFP in infected cells independently of the usage of antibodies. To this end, HFFs were infected with HD-UL52-mGFP and fixed 4 days later to be examined by immunofluorescence. The terminase proteins pUL56 and pUL89 as well as pUL44 (another marker for viral RCs) were visualized by specific antibodies, and samples were analyzed by confocal laser scanning microscopy. Figure 7 demonstrates that pUL52-mGFP was easily detectable in the infected cells, and moreover showed a distinct accumulation in viral RCs, where it co-localized with pUL44 as well as the terminase subunits. Although pUL52-mGFP was in part also detected outside of the RC as described previously (31), the vast majority was present inside of this subnuclear compartment. This finding revises our former observation on the pUL52 localization (31). In conclusion, the colocalization of pUL52 with pUL56 and pUL89 in the RC is consistent with the identified interactions of pUL52 with the terminase proteins.

### Terminase complex formation in the absence of pUL52

**Fig 8:**
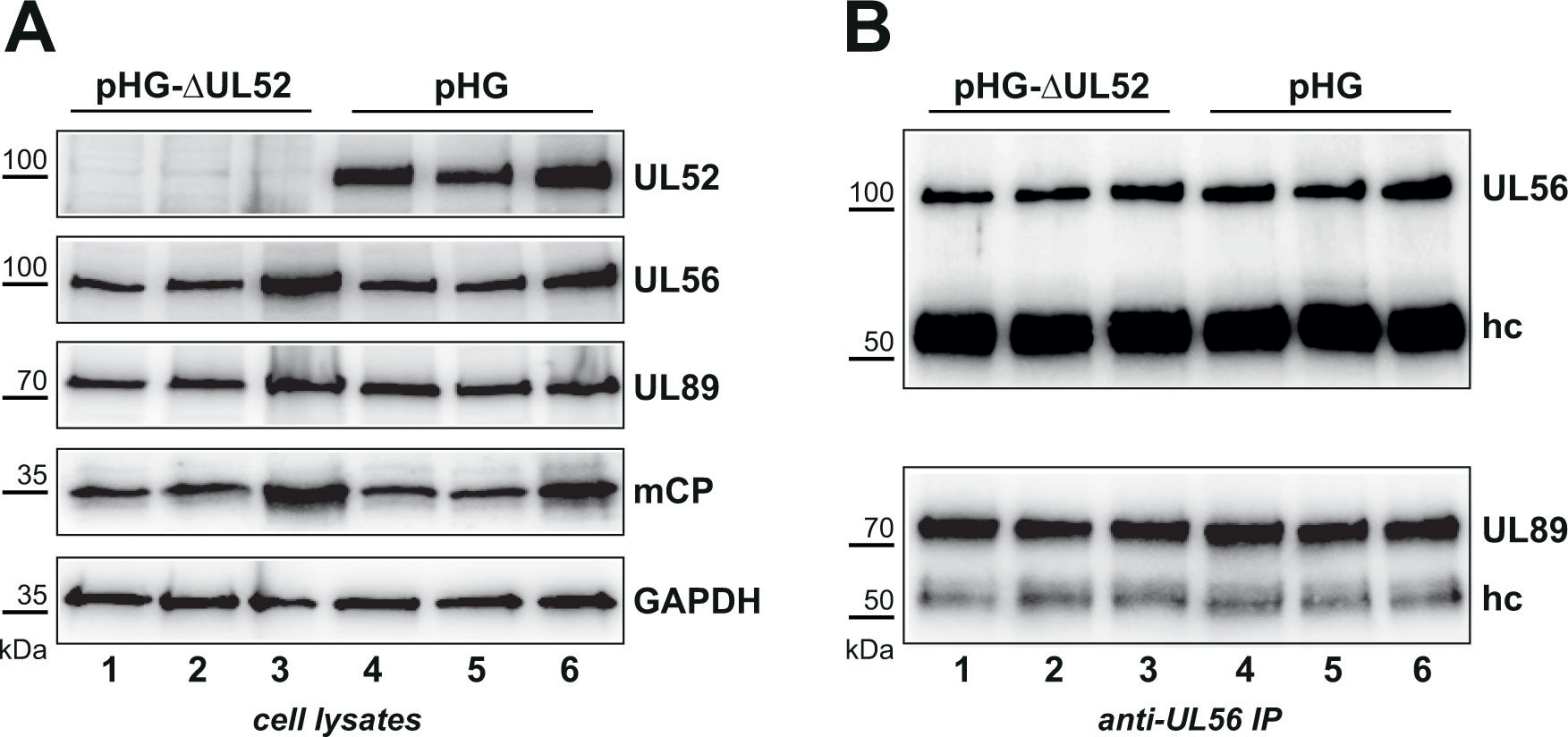
Expression and interaction of pUL56 and pUL89 in the absence of pUL52. (**A**) hTERT-RPE-1 cells were transfected by adenofection in triplicate with BACs pHG or pHG-ΔUL52, and cell lysates were prepared 4 days later. Whole cell lysates were examined by immunoblotting with antibodies against the proteins of interest (pUL52, pUL56, pUL89) as well as mCP as transfection control and cellular GAPDH as loading control. (**B**) Cell lysates shown in (**A**) were subjected to anti-UL56-IP, and bound proteins were investigated by immunoblotting, employing the pUL56 or pUL89 antibody. hc, antibody heavy chain (serving as loading control). Please note that the UL52 antibody exhibits weak cross-reactivity with an RPE-1-derived cellular protein that migrates with a similar apparent molecular mass as pUL52 (11, 12).

We have previously shown that the terminase components pUL51, pUL56, and pUL89 depend on each other regarding their stability and complex formation (11), i.e., in the absence of one subunit, the protein levels of the others are reduced and interaction between the remaining terminase subunits is impaired. Employing a UL52 deletion mutant, we reported that in the absence of pUL52, the terminase constituents still localize to viral RCs and binding of pUL56 to pUL89 did occur (31, 34). However, in those previous studies, we did neither investigate the levels of pUL56 and pUL89 when pUL52 was missing nor was the observed pUL56-pUL89 interaction assessed in direct comparison to the parental virus. We therefore now used the UL52 knock-out BAC pHG-ΔUL52 or the parental pHG genome (31) (Fig. 1) to transfect RPE-1 cells in triplicate by means of adenofection, a technique that allows the direct analysis of cells transfected with BAC genomes deleted for essential genes (34). Four days later, whole cell lysates were prepared and examined for pUL56 and pUL89 amounts by immunoblotting and subsequently subjected to Co-IP utilizing a UL56 antibody to test for pUL89 interaction. As shown in Fig. 8A, the lack of pUL52 had no influence on the levels of pUL56 or pUL89 (compare lanes 1–3 to lanes 4–6), and interaction among pUL56 and pUL89 was not disturbed in the absence of UL52 (Fig. 8B, compare lanes 1–3 to lanes 4–6). Because no suitable antibody is available, we could not test for pUL51 in this setting. Nevertheless, it is reasonable to deduce that incorporation of pUL51 into the terminase complex will proceed when pUL52 is missing, as pUL51 is needed to maintain appropriate protein levels of pUL56 and pUL89, and is moreover required to establish proper interaction between pUL56 and pUL89 (11). In sum, these results imply that pUL52 is not essential for assembly of the terminase.

## DISCUSSION

In this work, we identified several viral proteins associated with the HCMV UL52 protein that is required for viral genome cleavage and packaging. To our knowledge, this is the first description of pUL52-interacting proteins in HCMV-infected cells, thereby providing insight into the essential role of pUL52 in the viral infection cycle. Since complementary approaches were employed, we consider our results to be highly reliable. Furthermore, the discovered pUL52 interactors are consistent with the crucial function of pUL52 in viral genome encapsidation discovered in our previous study (31). We performed BioID proximity labeling as well as pull-down and Co-IP experiments using newly constructed HCMV mutants, for example, the ones expressing BioID2, an improved biotin ligase that is of smaller size than previously used BioID ligases, requires less biotin and also has enhanced labeling activity (64).

The most notable viral pUL52-interacting proteins discovered by MS and verified by immunoblotting were the terminase subunits pUL56 and pUL89 as well as the portal protein pUL104. Furthermore, our data indicate that pUL52 is in contact with viral proteins associated with viral DNA, among them were immediate early proteins IE1 and IE2, and the viral DNA polymerase accessory factor pUL44. These latter proteins were identified primarily via the BioID assay, while pull-down experiments argue against strong interaction. Since in the intact infected cells BioID labels adjacent proteins in their physiological environment, the observed association most likely resulted from the proximity of these proteins and of pUL52 to DNA and localization next to each other. Thus, streptavidin-mediated enrichment occurred because they got biotinylated, and not due to being connected via DNA after cell lysis, particularly since samples were treated with benzonase nuclease and denaturing buffers. The data therefore suggest that several viral DNA-associated proteins are located in the vicinity of pUL52 but do not form stable complexes.

Our data so far suggest the conjunction of pUL52 with viral genomes, yet we have previously shown that it is not necessary for viral genome replication (31). Applying sequence alignment and homology modeling, Muller and colleagues have recently described three conserved regions within the pUL52 C-terminus, which possibly form zinc fingers, pointing to a DNA-binding or oxidoreductase-like function (65). Mutation of aa residues characteristic of these motifs revealed their importance for growth of HCMV. Earlier, similar motifs have been identified in HSV-1 pUL32 as well as in KSHV ORF68 (36, 37). In fact, KSHV ORF68 was reported to adopt a homopentameric ring structure that binds DNA and displays nuclease activity (37, 38). These findings point to similar properties of pUL52, albeit binding of pUL52 to DNA remains to be experimentally proven.

Unlike the viral DNA-associated proteins IE1, IE2, and pUL44, the terminase subunits pUL56 and pUL89 were unequivocally identified by both the BioID and the pull-down assays, and likewise, the portal protein pUL104 was detected. Moreover, transient transfection experiments showed that pUL52 can interact with the terminase as well as with pUL104 in the absence of other viral proteins and of viral DNA. Taken together, these findings strongly argue for a role of pUL52 at the portal vertex, for instance, to support docking of the terminase to the portal. Although a previous report indicated that pUL56 binds to the portal, thereby coupling the terminase complex to the portal (14), this does not exclude a separate role of pUL52 in this process. This is in line with data obtained for α-herpesviruses, which showed that terminase-capsid binding is enhanced in the presence of HSV-1 pUL32 (the ortholog of HCMV pUL52), albeit pUL32 does not seem to be strictly needed for association of the terminase with capsids (66). Overall, these data support our hypothesis that pUL52 is binding to the terminase subunits as well as to pUL104, facilitating their interaction. Our MS data suggested that pUL51 is part of the pUL52-terminase protein complex as well, yet due to the lack of an appropriate pUL51-specific antibody, confirmation of the MS data by immunoblotting was not possible. Adding to that, this scenario is corroborated by our recent findings that pUL51, pUL56, and pUL89 are dependent on each other with respect to terminase assembly (11).

The results on pUL52 binding to the viral terminase were somehow surprising to us, as we had not observed this in a former study (10). Likewise, in a report using infection of cell lines expressing tagged versions of individual HCMV proteins, those viral proteins were not annotated as pUL52 interactors (67). To reassess our previously published findings, we now performed reciprocal Co-IP experiments by pull-down of pUL51, pUL56, or pUL89 using larger amounts of HCMV-infected cells than before, but again did not find evidence of pUL52 in the immunoprecipitated material (Fig. S3B). One explanation for this could be that only a portion of the pUL52 molecules interacts with pUL56 and pUL89, whereas a substantial fraction is probably associated with viral DNA, as has previously been shown for pUL52 orthologs (37, 38). Second, mapping of the UL56 and UL89 protein regions utilized for antibody generation (10) (or of the SF-tagged pUL51 N-terminus [10]) on a three-dimensional model of the terminase complex (based on the HSV-1 terminase subunits; [13]) revealed their localization near to each other (Fig. S4 (68)). It is therefore possible that the antibodies used during Co-IP compete with pUL52 for terminase binding, resulting in displacement of pUL52.

Confocal microscopy analyses disclosed that the terminase subunits did co-localize with pUL52 in nuclear RCs, which is consistent with our results showing their interaction in HCMV-infected cells. In an earlier study, we had reported that pUL52 is mainly nuclear but predominantly outside of the viral RC (31). Although seemingly contradictory, this finding can be explained by the use of a different antibody than before and of additional HCMV mutants generated in this work in which the BioID2-HA sequences are fused to pUL52. In the former HD-52HA mutant, the HA tag used for visualization of pUL52 was attached directly to the pUL52 N-terminus (31), whereas in the current HCMV mutants, the HA epitope is separated from pUL52 by 241 amino acids (corresponding to BioID2 and linker sequences). In this way, the HA tag at the very N-terminus of the BioID2-UL52 protein may be better accessible, thereby enabling its detection within the RC. In this respect, it is of note that recent data demonstrated that herpesviral RCs emerge through liquid-liquid phase separation, and that particularly, the late RCs are characterized by molecular crowding and increased viscosity, which may contribute to lowering the accessibility for antibodies within these structures (69, 70). Lastly, the HCMV mutant expressing a pUL52-mGFP fusion protein confirmed our revised findings on pUL52 subcellular distribution. Therefore, albeit pUL52 is also present at the nuclear periphery, it is mainly located inside the replication compartments, corroborating our data on pUL52-terminase interaction.

An obvious question is whether pUL52 acts as a sort of chaperone for the HCMV terminase, a valid hypothesis in view of our previous observation that pUL51, pUL56, and pUL89 are dependent on each other for mutual stabilization and interaction (11, 12). Hence, pUL52 could represent a fourth constituent of the terminase complex serving as a scaffold for terminase assembly. However, this does not seem to be the case, as in the absence of pUL52, the protein levels of the terminase subunits pUL56 and pUL89 were not reduced and their interaction was not impaired. As outlined above, we could not investigate pUL51 in this experiment. Nonetheless, given that pUL51 is needed to promote pUL56-pUL89 interaction and hence the formation and stability of the terminase complex (11), one can infer that, overall, formation of the heterotrimeric terminase complex does not require pUL52, although it may potentially foster oligomerization of the terminase into its presumed higher-order hexameric structure (13).

**Fig 9:**
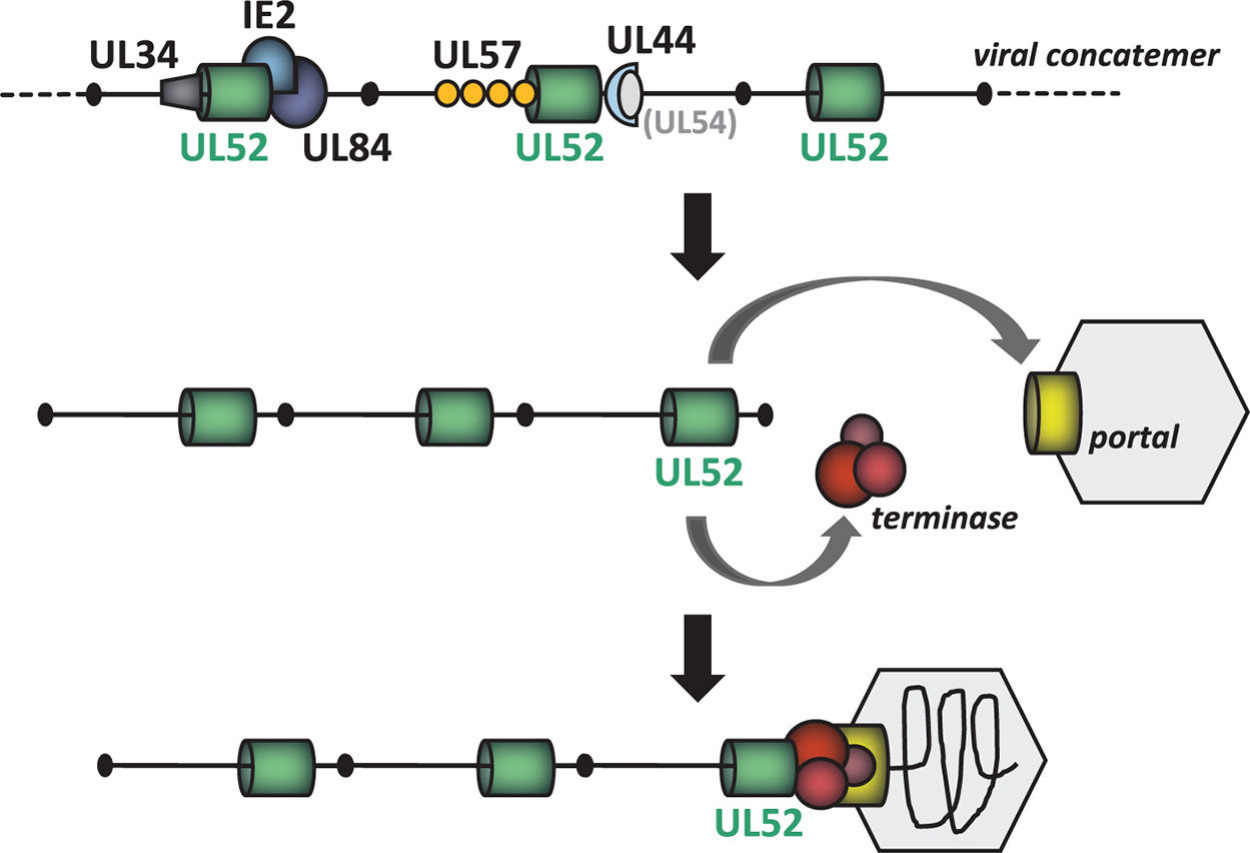
Model of pUL52 function in the course of the HCMV infection cycle. (Top) Based on BioID assays, pull-down studies, and cross-linking experiments, a scenario is proposed in which pUL52 is in proximity to or interacts with viral proteins associated with HCMV genomes. (Middle and bottom) The main task of pUL52 is to promote the association of viral DNA, the terminase complex, and the portal to initiate genome encapsidation and cleavage. Detected by BioID assay and confirmed by immunoblotting: IE2, UL44; identification by XL-MS: IE2, UL84, UL57, UL34, UL56, UL89; discovered in proximity labeling, pull-down assays, and XL-MS: terminase subunits UL56 and UL89. Portal protein UL104: identified by BioID assay as well as pull-down experiments and verified by immunoblotting. UL54 (DNA polymerase) is known to be in tight complex with UL44, yet was below threshold in the MS analyses.

Based on the results of this work, we developed a model in which pUL52 coordinates viral genome encapsidation (Fig. 9). In this respect, it is important to note that the different methodological approaches applied here are not identical, but complementary. The BioID assay mainly reveals spatial vicinity and is particularly suitable for detecting weak or transient interactions during the labeling period in infected cells, while pull-down experiments are rather indicative of stable interactions and formation of protein complexes at a given time point. Direct binding can be examined by XL-MS, which in turn requires the availability of lysine residues at the interfaces of the interacting proteins at a suitable distance. By combining these methods, a valid scenario of the corresponding protein-protein interactions can then be deduced. Proximity labeling indicated that pUL52 is adjacent to DNA-binding or chromatin-associated viral proteins (Fig. 9, upper part), and cross-linking points to close contact with IE2, pUL84, pUL57, and pUL34. IE2 and pUL84 form a complex which binds to the viral lytic origin of replication (oriLyt) to initiate viral DNA synthesis (58, 60, 71), and pUL57 (ssDNA-binding protein) and pUL44 (polymerase accessory factor) belong to the core viral replication machinery (1). The UL34 proteins also have binding sites near oriLyt (72), although their requirement for genome replication is controversial (73, 74). Interestingly, UL34 proteins were recently described to contribute to capsid maturation (74). While binding to viral DNA remains to be examined, it is reasonable to assume that pUL52 has this property, especially when considering the structural homology to the KSHV ortholog ORF 68 (37, 38). Keeping in mind that our previous experiments demonstrated that pUL52 is not required for HCMV DNA replication (31), we believe, however, that the association with DNA-binding proteins does not reflect the major task of pUL52 in HCMV-infected cells. Rather, we propose that the primary function of pUL52 is to orchestrate genome packaging and cleavage by bringing together the viral DNA, the terminase complex, and the portal (Fig. 9). Our findings deepen the understanding of this essential process of the HCMV infection cycle. Moreover, the knowledge of UL52 interaction partners will allow to set up specific screening approaches to identify small molecule inhibitors that can disrupt the viral genome encapsidation network, thereby opening up new perspectives for the development of effective antivirals against HCMV and potentially also other herpesviruses.

## Data Availability

All mass spectrometry proteomics raw data have been deposited to the ProteomeXchange Consortium via the PRIDE (75) partner repository with the dataset identifier PXD060825.

## SUPPLEMENTAL MATERIAL

Fig. S1 jvi.02201-24-s0001.pdf Action of benzonase and gene ontology analysis.

Fig. S2 jvi.02201-24-s0002.pdf Genome structures of mutant HCMV BACs.

Fig. S3 jvi.02201-24-s0003.pdf Reciprocal co-IPs.

Fig. S4 jvi.02201-24-s0004.pdf Predicted terminase structure.

Table S1 jvi.02201-24-s0005.docx Oligonucleotides.

Table S2 jvi.02201-24-s0006.xlsx Cellular pUL52 interaction partners.

ASM does not own the copyrights to Supplemental Material that may be linked to, or accessed through, an article. The authors have granted ASM a non-exclusive, world-wide license to publish the Supplemental Material files. Please contact the corresponding author directly for reuse.
