# Supplementary material for: Interaction of human cytomegalovirus pUL52 with major components of the viral DNA encapsidation network underlines its essential role in genome cleavage-packaging
Source: J Virol. 2025 Mar 10;99(4):e02201-24. doi: 10.1128/jvi.02201-24 (PMC11998523; doi:10.1128/jvi.02201-24)
Supplement: Fig. S1 — Action of benzonase and gene ontology analysis. [file jvi.02201-24-s0001.pdf]

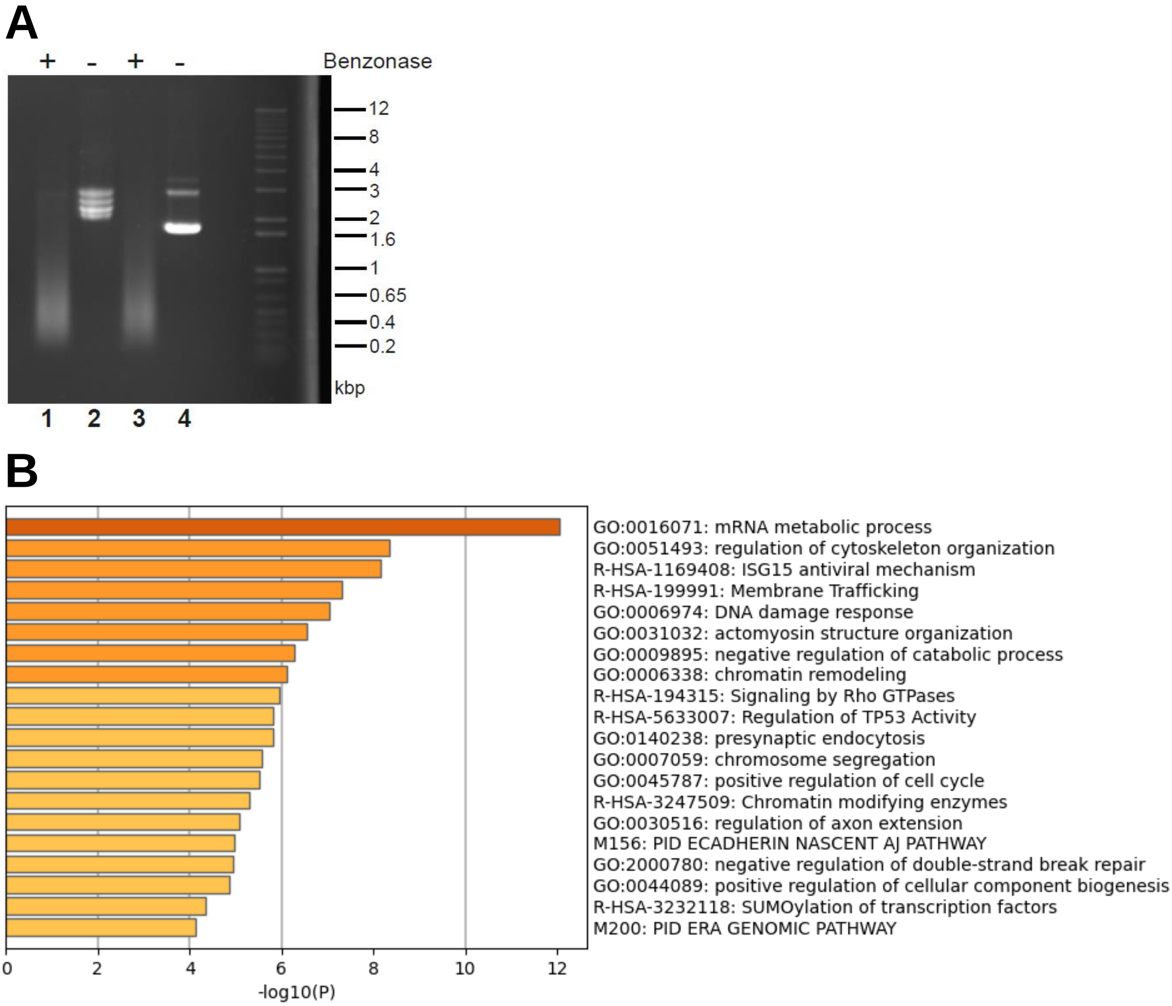

Supplementary Figure S1 (A) Action of benzonase under the experimental conditions used. pUC19 plasmid DNA was mixed with cell lysate (lanes 1 and 2) or lysis buffer (lanes 3 and 4), both containing protease inhibitors. Benzonase was then either added or omitted, and samples were analyzed by agarose gel electrophoresis and ethidium bromide staining. Please note that the appearance of several bands exhibiting a slightly different migration pattern in lane 2 is owed to the different composition in ionic strength and the presence of proteins in the cell lysate samples. (B) Functional categories of host proteins enriched by BioID assay. Please see Materials and Methods section for further details.
