# Supplementary material for: Interaction of human cytomegalovirus pUL52 with major components of the viral DNA encapsidation network underlines its essential role in genome cleavage-packaging
Source: J Virol. 2025 Mar 10;99(4):e02201-24. doi: 10.1128/jvi.02201-24 (PMC11998523; doi:10.1128/jvi.02201-24)
Supplement: Fig. S2 — Genome structures of mutant HCMV BACs. [file jvi.02201-24-s0002.pdf]

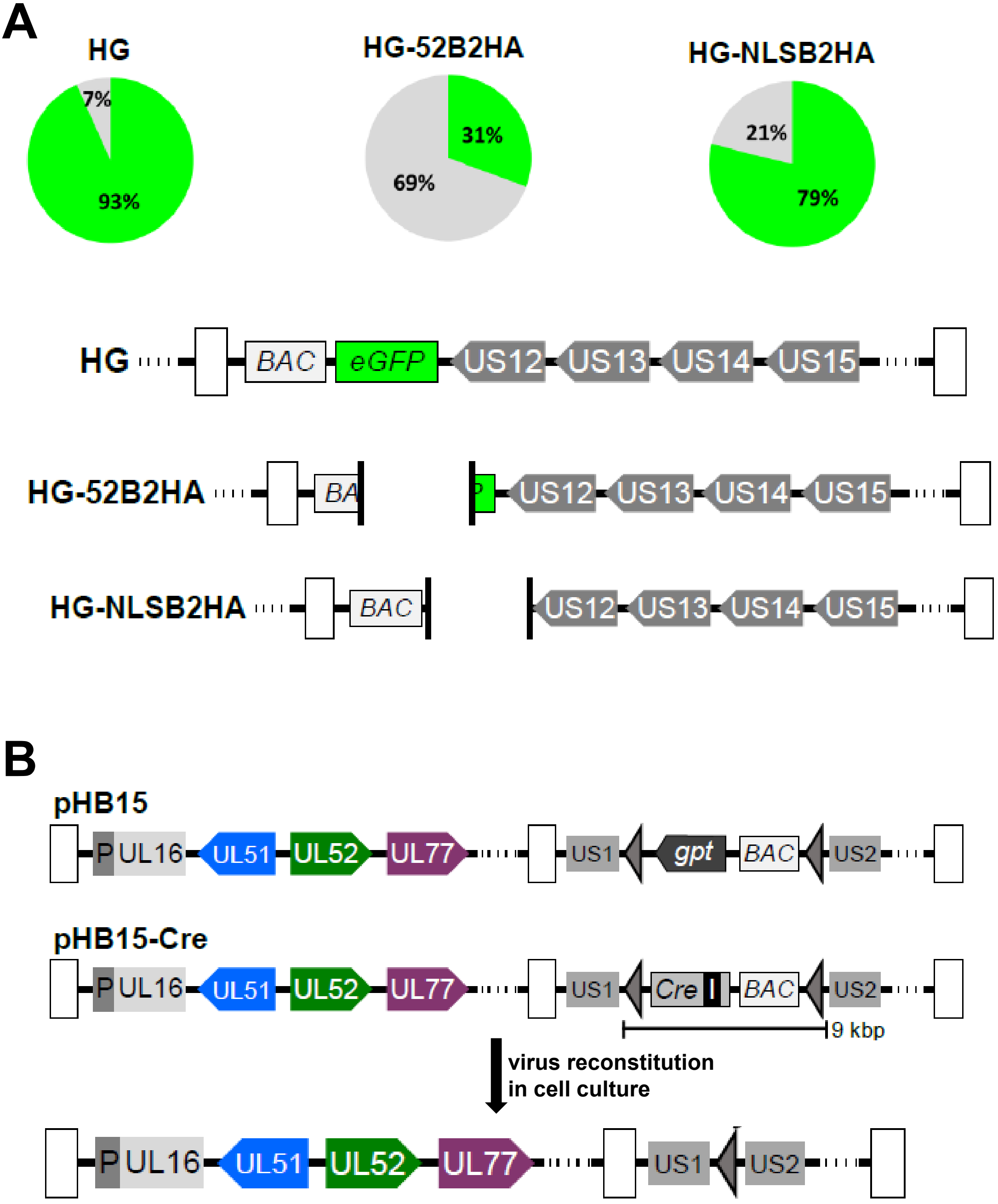

Supplementary Figure S2 Deletions in and adjacent to the BAC vector sequences observed with HCMV-GFP-based BioID2 mutants and construction of pHB15-based BioID2 mutants with self-excisable BAC vector. (A) Upper part, fraction of green fluorescent plaques observed with the HCMV-GFP-BioID2 mutants or the parental virus after virus reconstitution from the respective BAC genomes. Total plaques were counted following GIEMSA staining. Lower part, schematic representation of the prevalent deletions detected in the US region of the indicated viral genomes (drawing not to scale). Viral DNA was isolated from virus particles present in the supernatant of infected HFF. (B) Schematic overview of pHB15 and pHB15-Cre genomes. The *gpt* gene in pHB15 (top) was replaced with a Cre recombinase gene harboring an intron (I), yielding BAC pHB15-Cre (middle). Upon transfection of pHB15-Cre-derived BAC genomes the BAC vector together with the Cre cassette is excised via the flanking *loxP* sites (bottom).
