# Supplementary material for: Interaction of human cytomegalovirus pUL52 with major components of the viral DNA encapsidation network underlines its essential role in genome cleavage-packaging
Source: J Virol. 2025 Mar 10;99(4):e02201-24. doi: 10.1128/jvi.02201-24 (PMC11998523; doi:10.1128/jvi.02201-24)
Supplement: Fig. S3 — Reciprocal co-IPs. [file jvi.02201-24-s0003.pdf]

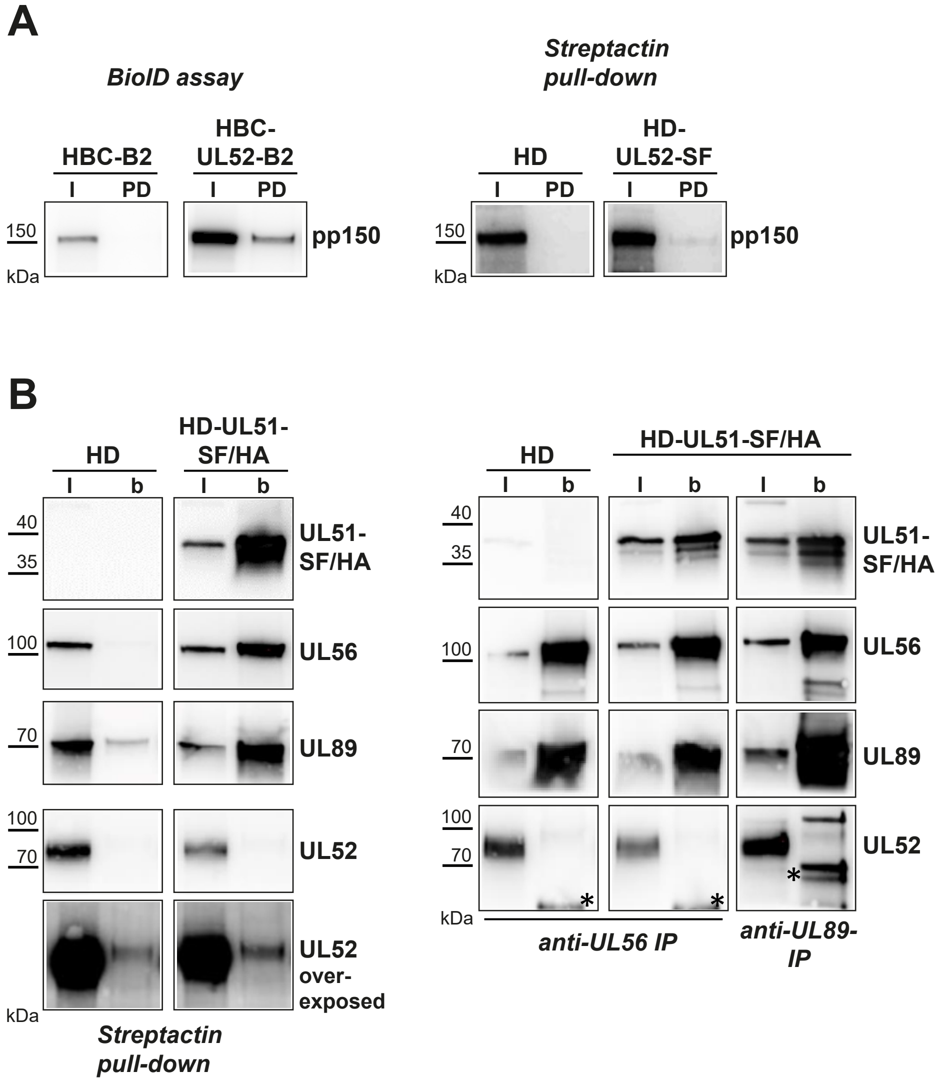

Supplementary Figure S3 (A) Evaluation of pUL32 (pp150) as potential pUL52 interactor by BioID assay (left) and Streptactin pull-down (right). I, input samples, PD, pulled down material. (B) Assessment of pUL52-terminase interaction by reciprocal Co-IP. HFF were infected with the indicated viruses (HD or HD-UL51-SF/HA), and 4 days later, cell lysates were prepared and subjected to Streptactin pull-down or IP employing either the UL56- or UL89-specific antibody. Cell lysates (I, input) and pulled-down material (b, bound) was analyzed by immunoblotting using the indicated antibodies (detection of pUL51-SF/HA was performed utilizing an anti-HA antibody). Left: Streptactin pull-down strongly enriched pUL51-SF/HA as well as the terminase subunits pUL56 and pUL89, while this was not observed for pUL52. HD expressing untagged pUL51 served as control virus. Right: Neither anti-UL56 IP from HD- or HD-UL51-SF/HA-infected cells, nor anti-UL89 IP from HFF infected with HD-UL51-SF/HA revealed association of pUL52 with the terminase subunits. Conversely, as expected, complex formation of the terminase constituents pUL56, pUL89 and pUL51 was readily seen (please note that pUL51 expressed by HD is not detected by the anti-HA antibody, upper left panel). Asterisks: signals presumably originating from antibody heavy chains.
