# Supplementary material for: Interaction of human cytomegalovirus pUL52 with major components of the viral DNA encapsidation network underlines its essential role in genome cleavage-packaging
Source: J Virol. 2025 Mar 10;99(4):e02201-24. doi: 10.1128/jvi.02201-24 (PMC11998523; doi:10.1128/jvi.02201-24)
Supplement: Fig. S4 — Predicted terminase structure. [file jvi.02201-24-s0004.pdf]

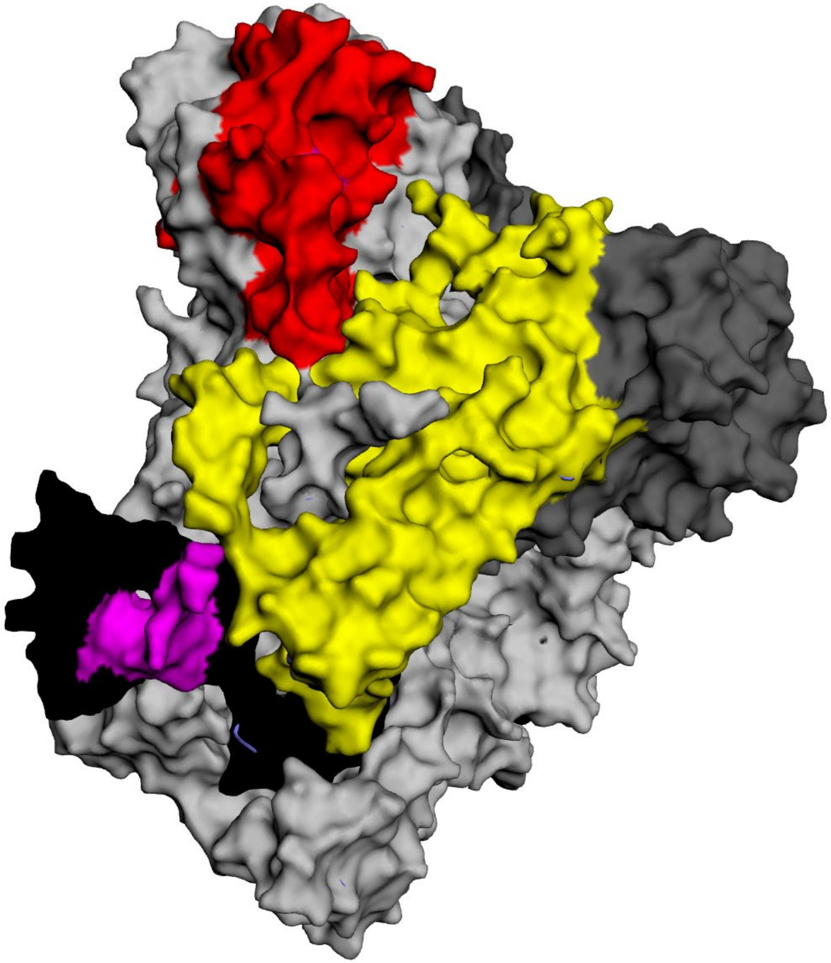

Supplementary Figure S4 Mapping of the HCMV pUL56 and pUL89 fragments employed for antibody generation and of the pUL51 N-terminus on the three-dimensional model of the HSV-1 terminase (13). Red, pUL56 C-terminal part (conserved with respect to its HSV-1 ortholog pUL28); yellow, pUL89 N-terminal region (conserved regarding its HSV-1 ortholog pUL15); pink, pUL51 N-terminus (corresponding to the respective part of HSV-1 pUL33). Remaining residues of HSV-1 pUL28, pUL15, and pUL33 are depicted in light and dark grey, and black, respectively. The model was drawn using EzMol (74) (http://www.sbg.bio.ic.ac.uk/ ezmol/).
