# Supplementary material for: Interaction of human cytomegalovirus pUL52 with major components of the viral DNA encapsidation network underlines its essential role in genome cleavage-packaging
Source: J Virol. 2025 Mar 10;99(4):e02201-24. doi: 10.1128/jvi.02201-24 (PMC11998523; doi:10.1128/jvi.02201-24)
Supplement: Table S1 — Oligonucleotides. [file jvi.02201-24-s0005.docx]

**Supplementary Table S1** Oligonucleotides used for plasmid cloning and BAC mutagenesis.

| **Name** | **Sequence (5’ > 3’)** |
| --- | --- |
| pUC-BioID.for | agctcggtacccggggatccatg*tacccttacgatgtaccggattacgca*ggagctagcttcaagaacctgatctgg |
| pUC-BioID.rev | gaccatgattacgccaagcttgctagctccgcttcttctcaggctgaac |
| BioID-Kn.for | cgcctgcagctgcccctggtgctgggccacgcatcgtggccggatc |
| BioID-Kn.rev | cgcctgcagcaggttctcgaactccttggtgaccacgtcgtggaatg |
| 6K-52RFP.for | P~gatatcaatccgagtacccacgtgagca |
| 6K-52RFP.rev | P~ggcgatcggcgccgctgatt |
| UL52-SF.for | cgcgatatcccaccatggattataaagatg |
| UL52-SF.rev | cgcgatatcatcctctccgctagctcc |
| UL52-SF-insert.for | ctggctagcgtttaaacttacccaccatggattataaag |
| UL52-SF-insert.rev | cctctagactcgagcctagacatacttgtctatcac |
| UL51-HA.for | ctggctagcgtttaaacttaccaccatgtacccatacgacgtcccag |
| UL51-HA.rev | cctctagactcgagcttatttacccggcgccgac |
| UL104.for | ggctagcgtttaaacttaagcttccaccatggagcgaaaccactgg |
| UL104.rev | cctctagactcgagcggccgcctagtgaaatccgtatggacc |
| UL52-mGFP.for | cgcgatatcccaccatggtgagcaagggcga |
| UL52-mGFP.rev | cgcgatatcgctggccgaccccttgtacagctcgtccatgcc |
| UL52-BioID2.for | cttttttccgcgtcctcaatcagcggcgccgatcgccatgtacccttacgatgtaccgga |
| UL52-BioID2.rev | gagtcgttgggccgttactgctcacgtgggtactcggattactaccgccgctacctccgcttcttctcaggctgaact |
| BioID2-NLS-Kon.for | tatgaggagcgcgcacaaaggaccgttaggcgccggcatg**ccgaagaagaagcgaaaggtc**tacccttacgatgtaccgga |
| BioID2-NLS-Kon.rev | agcgtgaacgttgcacgtggcctttgcggttatccgttcagcttcttctcaggctgaact |

italics: HA tag; underlined: linker sequence; bold: SV40 NLS; P~: oligonucleotides phosphorylated at the 5’-end.
